# Electrochemical Water Oxidation Mechanisms Involving Macrocyclic Copper(II) Complexes: Ligand Ring Size Effects on Catalytic Cycles

**DOI:** 10.1002/cphc.202500637

**Published:** 2025-12-10

**Authors:** João Pedro C. S. Neves, Roberto Rivelino, Tiago Vinicius Alves, Vitor H. Menezes da Silva

**Affiliations:** ^1^ Departamento de Físico‐Química Instituto de Química Universidade Federal da Bahia Salvador Bahia 40170‐115 Brazil; ^2^ Instituto de Física Universidade Federal da Bahia Salvador Bahia 40210‐340 Brazil

**Keywords:** catalyst design, computational chemistry, density functional theory, electrocatalysis, water oxidation

## Abstract

A key challenge of electrocatalytic water oxidation for H2 production remains in modulating structural and electronic features of transition metal complexes to enhance catalytic performance. Herein, inspired by previous experimental and computational studies on the macrocyclic catalyst [Cu(14‐TMC)]^2+^ (1,4,8,11‐tetramethyl‐1,4,8,11‐tetraazacyclotetradecane), we present a theoretical investigation based on Density Functional Theory (DFT) to examine the mechanistic impacts of its ring size reduction. To this end, we evaluated the water oxidation catalytic cycle mediated by [Cu(12‐TMC)]^2+^, providing a comprehensive analysis of the electrochemical oxidation, O—O bond formation, and O_2_ evolution steps. Subsequently, we compare mechanistic features of [Cu(14‐TMC)]^2+^ and [Cu(12‐TMC)]^2+^ highlighting similarities and differences in the key reaction routes and intermediates, revealing that ligand ring size affects the electronics, steric hindrance and, consequently, the coordination numbers of these species. Notably, the rate‐determining step of both catalytic cycles is the O—O bond formation exhibiting significant differences in their mechanisms, especially regarding the structures of key intermediates. Despite that, both mechanisms have comparable energy barriers. For instance, the Gibbs free energy barriers are computed to be 18.96 and 19.26 kcal/mol for [Cu(12‐TMC)]^2+^ and [Cu(14‐TMC)]^2+^ catalysis, respectively. However, [Cu(12‐TMC)]^2+^ provided more intricate mechanisms due to being more susceptible to ligand reorganization in the Cu coordination sphere.

## Graphical abstract

The ring size of the ligand strongly influences electronic properties, steric effects, and coordination modes of key intermediates. Both catalysts show similar energy barriers for O—O bond formation, but [Cu(12‐TMC)]^2+^ exhibits more complex mechanisms due to ligand reorganization.

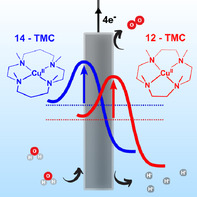

## 1 Introduction

The promise of a sustainable future deeply lies on the energy transition,^[^ ^1^ ^]^ and one of the most promising contenders to replace fossil fuels as a clean and renewable alternative is the hydrogen gas, H_2_.^[^ ^2^ ^]^ This resource can be obtained through several processes such as the solar‐driven electrolysis of water (Equation (1)).^[^ ^3^, ^4^ ^]^ The overall reaction is, however, thermodynamically demanding. Besides, in order to generate the other product, O_2_, along with the O—O bond formation, four electrons and protons are required to be released from two water molecules, which thus poses a complex mechanistic scenario.^[^ ^5^, ^6^ ^]^

(1) $${\text{2H}}_{2}O\to O_{2}+{\text{2H}}_{2}\;{E{}^{\circ}}_{\text{SHE}}=-1.23\;V$$

This kinetic drawback is the main reason why recent research has been focusing on increasingly better catalysts for the water oxidation (WO),^[^ ^7^ ^]^ i.e., the anodic half‐reaction of Equation (1), outlined above. The field of homogeneous catalysis, mainly centered on transition metal complexes,^[^ ^8^, ^9^, ^10^, ^11^ ^]^ has yielded substantial results as well as insights about the mechanism in question, especially with the support of theoretical investigations.^[^ ^12^, ^13^, ^14^, ^15^, ^16^ ^]^ Although the earliest satisfactory catalytic activities were achieved by noble metals like Ir and Ru,^[^ ^17^, ^18^ ^]^ the current focus of studies on earth‐abundant transition metal complexes is building up with promising catalyst candidates.^[^ ^19^, ^20^, ^21^ ^]^

Among a wide variety of first‐row transition metals explored, copper (Cu) has been shown to be favorable due to low overpotentials and good electrochemical stability in both neutral and alkaline conditions.^[^ ^22^ ^]^ This significant electrocatalytic activity is, in part, attributed to the lability with which the Cu metallic center ranges from coordination to decoordination of ligands.^[^ ^23^ ^]^ Furthermore, the conformational complexity of the ligand moiety is also a feature to be considered for Cu‐based WO catalysts. Several ligands were extensively investigated in many architectures, such as pyridine,^[^ ^24^, ^25^, ^26^, ^27^ ^]^ amide,^[^ ^28^, ^29^ ^]^ porphyrin,^[^ ^30^ ^]^ and peptide types;^[^ ^31^ ^]^ moreover, dinuclear complexes were reported as well.^[^ ^32^, ^33^, ^34^, ^35^ ^]^ It has been evidenced that the coordination environment plays a key role in the mechanism, since it changes the structure of the metal‐containing reaction intermediates, which are involved in the reaction pathway, and, consequently, their respective reaction energy barriers. Hence, the major aim of transition metal WO's homogeneous catalysis resides in the design of ligands in order to improve the catalytic properties.

**Scheme 1:**
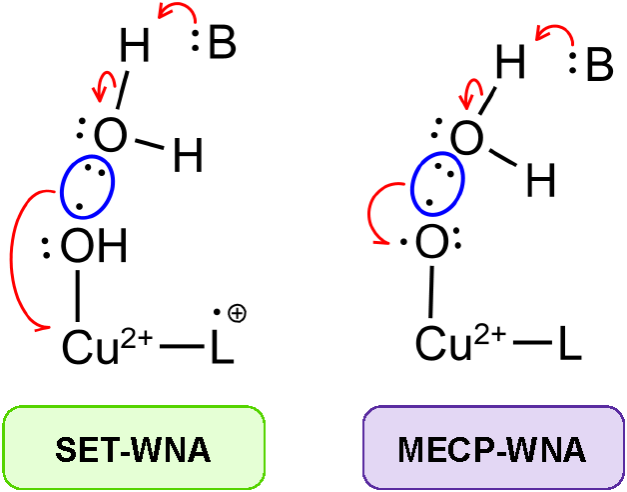
Possible O—O bond formation routes investigated in [Cu(14‐TMC)]^+2^ catalysis (14‐TMC is labeled as L). Here, B is a basic (proton acceptor) species in solution; its participation in the mechanism may be concerted or not.

Recently, the macrocyclic complex [Cu(14‐TMC)]^+2^ (14‐TMC = 1,4,8,11‐tetramethyl‐1,4,8,11‐tetraazacyclotetradecane) has been tested as a catalyst for WO.^[^ ^36^ ^]^ The mechanism of the WO electrocatalytic cycle was theoretically investigated^[^ ^37^ ^]^ and divided into three main parts: first, an endergonic electrochemical activation is required to drive the subsequent O—O bond formation, and then the O_2_ is released as a result of highly exergonic electrochemical reactions. The O—O bond formation was shown as the rate‐determining step. Two O—O bond formation mechanisms have been proposed: water nucleophilic attack (WNA), which consists of a Single Electron Transfer (SET) into the metallic center (this process is also referred to as SET–WNA). In addition, given the multiconfigurational character of such complexes,^[^ ^38^ ^]^ the connection between oxygens may also occur through a Minimal Energy Crossing Point (MECP) (also referred to as MECP–WNA). Both pathways are depicted in **Scheme** **1**, highlighting the auxiliary role of the proton acceptor (B). In this case, HPO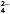 anion present in solution (pH = 7 with phosphate buffer) improves the nucleophilic character of the attacking water.^[^ ^39^, ^40^, ^41^, ^42^, ^43^ ^]^

The 14‐TMC ligand makes up a family of alkyl–amine macrocycles; they can vary in size and/or can display different functional groups attached to both carbon and nitrogen.^[^ ^44^ ^]^ With other ion metallic centers, some of their counterparts Tetramethyl‐cyclam (TMC) ligands were also studied for WO, revealing catalytic activity.^[^ ^45^, ^46^, ^47^ ^]^ In the case of [Ni(12‐TMC)]^2+^ (12‐TMC = 1,4,7,10‐tetramethyl‐1,4,7,10‐tetraazacyclododecane), for example, similar experimental conditions of [Cu(14‐TMC)]^2+^ catalysis (pH = 7 and phosphate buffer) were also employed. The applications of complexes with alkyl–amine macrocycles are widely known in other branches of molecular catalysis, as shown for C–H activation, wherein the ring size modification in the macrocycle leads to considerable impacts on electronic configuration and steric hindrance of the ligand.^[^ ^48^, ^49^, ^50^, ^51^ ^]^

**Scheme 2:**
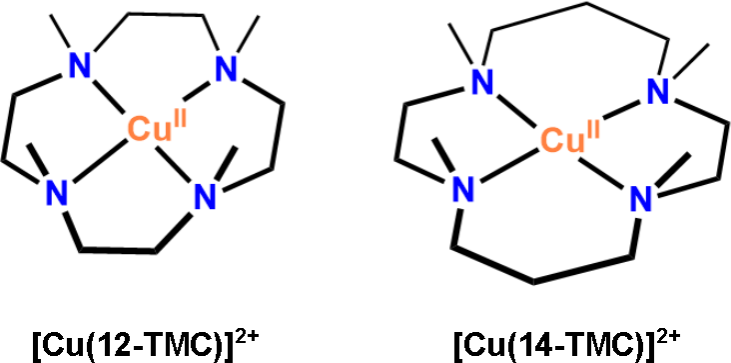
Structures of the catalysts [Cu(12‐TMC)]^2+^ and [Cu(14‐TMC)]^2+^.

In the present work, we investigate the catalytic performance of the complex [Cu(12‐TMC)]^2+^ (**Scheme** **2**) as a WO catalyst. Specifically, we computationally investigated, using electronic structure methods, the WO electrocatalytic steps, showing the possibility of Cu‐containing reaction intermediates in several coordination fashions. The kinetics and thermodynamics of this WO catalysis were obtained and discussed, showing their impacts on the mechanisms of the catalytic cycle steps. In order to give a more detailed picture, a computational comparison of 12‐TMC and 14‐TMC catalyses is presented. We believe that the comprehensive study shown here on the catalyst [Cu(12‐TMC)]^2+^, a size‐reduced 14‐TMC Cu(II) complex, might shed light on the design and modelling of new TMC macrocyclic ligands with enhanced activity toward WO.

## 2 Results and Discussion

### 2.1 Mechanisms of WO Catalysis with [Cu(12‐TMC)]^2+^

#### 2.1.1 Electrochemical Activation

**Figure 1:**
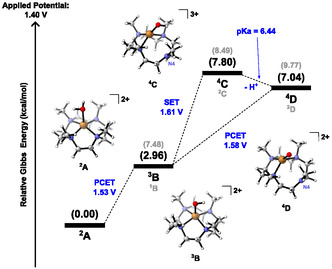
Free energy diagram of the complex ^2^ **A**'s electrochemical activation at 298.15 K and pH = 7, with an applied potential of 1.4 V.

Our theoretical screening of the WO mechanism catalyzed by the complex [Cu(12‐TMC)]^2+^ begins with the electroactivation of the catalyst (**Figure** **1**). For instance, in the case of [Ni(12‐TMC)]^2+^ catalysis, a hexacoordinated (octahedral) complex with two water molecules around nickel's vicinity was revealed.^[^ ^47^ ^]^ In contrast, in the case of the copper complex, we could not locate a similar Cu complex structure with a coordination number of six, since one of the water molecules tends to decoordinate from the Cu center, thus forming a distorted square pyramid. For that reason, the catalytic activation by electrochemical oxidation starts at the water‐coordinated complex, [Cu(12‐TMC)H_2_O]^2+^ (labeled as ^2^ **A**); from there, as occurs in the 14‐TMC coordination environment, an endergonic Proton‐Coupled Electron Transfer (PCET), with a potential computed to be 1.53 V, leads to the triplet species ^3^ **B**, whose singlet state lies 4.52 kcal/mol above, as depicted in Figure 1. Spin density analysis (see Section S2 of Supporting Information, which presents all spin density values) from ^2^ **A** to ^3^ **B** shows unpaired electrons located between the copper (mostly) and oxygen atoms, and also on the ligand.

In sequence, two kinds of electrochemical events related to the catalyst activation may occur: a SET (1.61 V) or a PCET (1.58 V), leading to ^4^ **C** and ^4^ **D**, respectively, both connected by a proton loss with pK_a_ of 6.44 (Figure 1). Both intermediates display an uncoordinated nitrogen (N4) and have the quartet as the most stable multiplicity state, while the doublet states lie, respectively, 0.69 and 2.73 kcal/mol above. Spin density population analyses reveal that, in either case (quartet or doublet states), N4 holds an unpaired electron (and thus a formal charge of +1), so that the copper ion remains in the oxidation state of +2. Hence, these structures demonstrate the noninnocent nature of the ligand (redox activity). Additionally, other possible conformations of **C** and **D** were also investigated (their energies are presented in Figure S7, Supporting Information). However, these conformers lie energetically above the lower energy structures depicted in Figure 1.

#### 2.1.2 O—O Bond Formation

**Figure 2:**
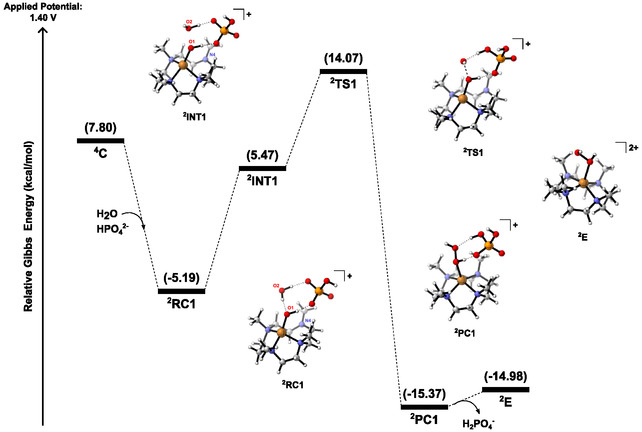
Gibbs energy diagram at 298.15 K for the O—O bond formation throughout pathway ^4^ **C**.

The formation of a σ‐type chemical bond between two oxygens can proceed through two mechanisms from ^4^ **C** or ^4^ **D**, both formed as a result of the electrochemical activation. Starting with the reaction pathway related to ^4^ **C** (**Figure** **2**), the approach of a water molecule and a HPO_4_^2−^ anion to the complex results in a significant stabilization of the system in doublet multiplicity (^2^ **RC1**). Spin density investigation of this intermediate shows that the Cu ion stays in oxidation state of +3, while the oxygen holds its electron pairs, resulting in a formal charge of −1. Indeed, this configuration maximizes the interaction of HPO_4_^2−^ with the coordinated OH, which explains the lower energy shown for ^2^ **RC1** in Figure 2.

**Figure 3:**
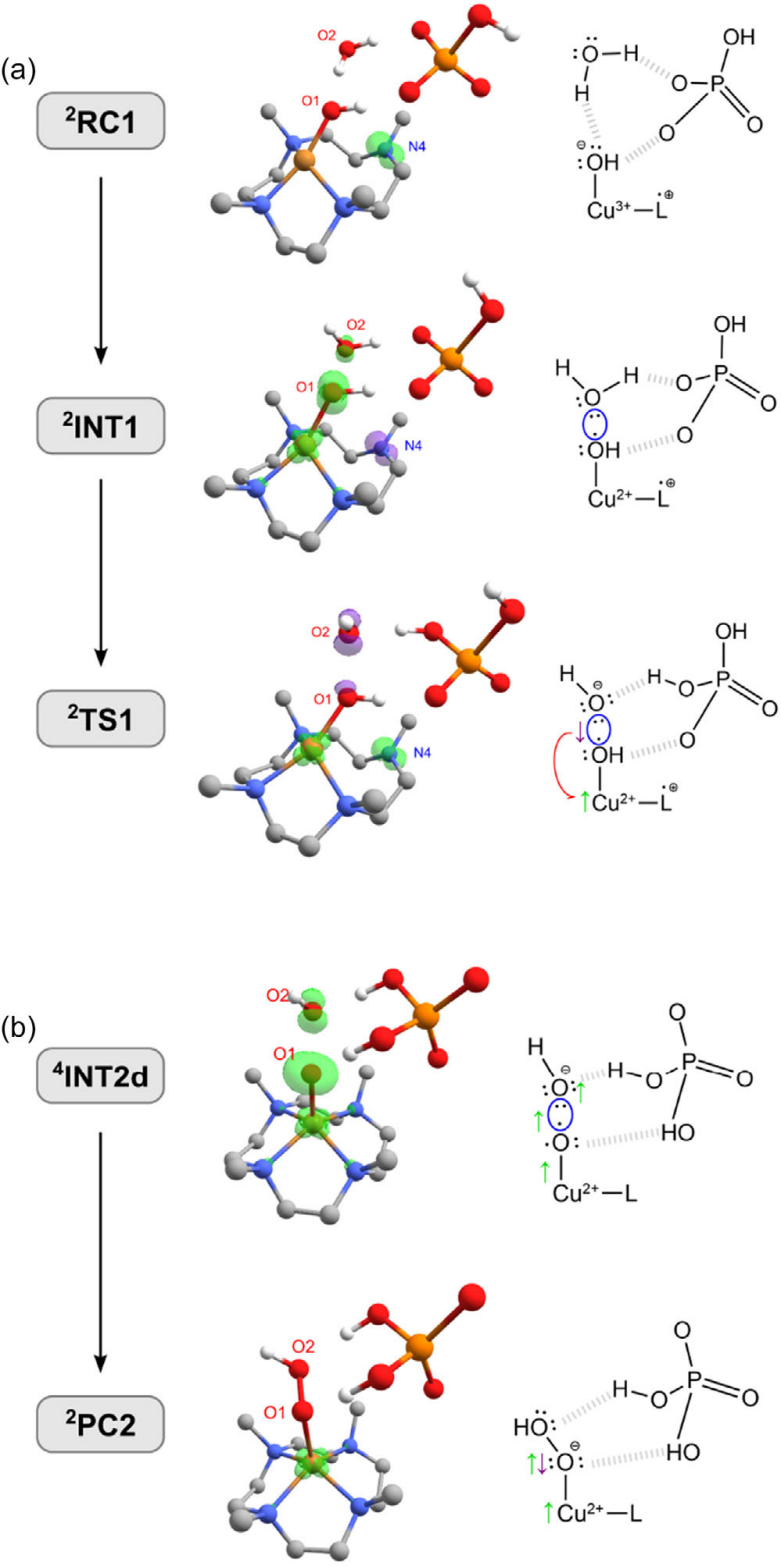
Spin densities of selected structures in pathways a) ^4^ **C** and b) ^4^ **D**, illustrating the O—O bond formation through a SET–WNA and a MECP—WNA mechanism, respectively. H atoms attached to carbon were omitted for clarity.

Furthermore, another potential prereactive complex intermediate is ^2^ **INT1**, which was found lying 10.66 kcal/mol above ^2^ **RC1**. The electronic configuration of ^2^ **INT1** resembles that of the doublet ^2^ **C** (See **Figure** **3**, S7, Supporting Information). Spin densities showed that the Cu center in ^2^ **INT1** exhibits an oxidation state of +2, with the oxygen holding one unpaired electron. In fact, this unpaired electron is shared between O1 and O2, thus resulting in a three‐electron σ bond with antibonding character.^[^ ^52^, ^53^, ^54^ ^]^ Subsequently, we identified a transition state (TS) for the O—O bond formation (^2^ **TS1**) wherein the copper displays a shift to an oxidation state of +1. Intrinsic reaction coordinate (IRC) calculations revealed a concerted proton abstraction by the HPO_4_^2−^ anion; indeed, ^2^ **RC1** has been shown to be the proper minima connected to ^2^ **TS1**. However, the direct connection between ^2^ **RC1** and ^2^ **TS1** implies a classic WNA, which is not likely to happen due to the absence of a Cu–O double bond nature. In the presence of an oxo ligand, the π donation from the copper increases the electrophilicity of the oxygen, which is also a π donor, so that the antibonding character prevails. Hence, in the case of copper, a transition metal situated far beyond the “oxo wall”, the Cu=O bond character is less evident.^[^ ^55^, ^56^, ^57^, ^58^, ^59^ ^]^ Based on this, it is much more plausible that the O—O bond formation proceeds via a SET process from ^2^ **INT1** through ^2^ **TS1**, as depicted in Figure 3a. This mechanistic scenario involving ^2^ **RC1**, ^2^ **INT1,** and ^2^ **TS1**, therefore, implies shifting the oxidation state of the copper center.

**Figure 4:**
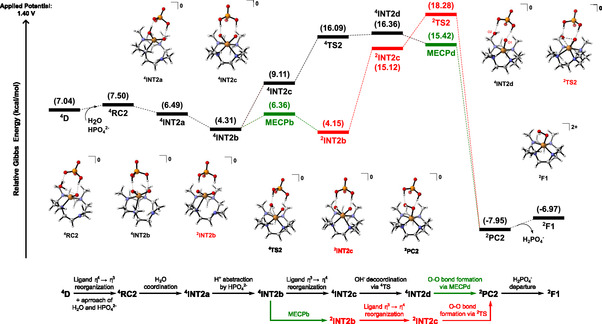
Gibbs energy diagram at 298.15 K for the O—O bond formation throughout pathway ^4^ **D**.

Moving now on the pathway related to ^4^ **D** (**Figure** **4**), the introduction of a water molecule and an HPO_4_^2−^ anion leads to a quartet intermediate, ^4^ **RC2**. At this point, the reduced ligand size of 12‐TMC compared to 14‐TMC (Scheme 2) allows the coordination of another water to Cu, i.e., the water is introduced into the coordination sphere of the complex, resulting in the distorted pentacoordinate environment of ^4^ **INT2a**. Spin population densities (Figure S5, Supporting Information) exhibit the copper in an oxidation state of +2 and one nitrogen situated outside the coordination sphere, which carries a formal charge of +1; thus, this uncoordinated nitrogen possesses an unpaired electron. In sequence, the HPO_4_^2−^ anion accepts the proton from the water, leading to ^4^ **INT2b**. Unfortunately, we were not able to locate a TS related to this proton abstraction. Even after performing a relaxed scan varying the O—H bond distance in order to find a guess structure for this TS, no saddle point was located. Hence, we presume that this step is barrierless, showing that ^4^ **INT2b** can bifurcate into two possible routes.

The first route begins with a reorganization in the coordination sphere, shifting from a pentacoordinate to a hexacoordinate environment. This exchange in the ligands, which is energetically feasible, results in ^4^ **INT2c**, wherein the oxidation state of the copper is +3. Afterward, a new mechanistic scenario was revealed by the density functional theory (DFT) calculation regarding the 12‐TMC ligand. The TS (^4^ **TS2**) was located and IRC calculations showed the ${\text{OH}}^{-}$ ion moving away from the Cu center until reaching ^4^ **INT2d**. This is equivalent to an electron transfer from the oxygen O1 to the copper, which shifts the oxidation state to +2 (Figure S9, Supporting Information); in other words, the complex ranges from side‐on to end‐on O–O configuration,^[^ ^51^ ^]^ and this process is computed to be potentially reversible. However, the connection between both oxygen atoms should take place through a MECP (**MECPd**, Figure S8, Supporting Information), leading to the complex ^2^ **PC2** in a doublet state, which is considerably more stable; thus, a positive driving force for this O—O bond formation takes place via this reaction mechanism. Figure 3b depicts the electron density transfer from O2 to O1, which is supported by the spin density analysis and by both Hirshfeld and Voronoi population charges. See details of these data grouped in the Supporting Information, Section S4, Supporting Information.

The second route from ^4^ **INT2b** arises from MECP between ^4^ **INT2b** and ^2^ **INT2b**, named **MECPb** (Figure S8, Supporting Information). Following **MECPb**, the intermediate ^2^ **INT2b** on the doublet surface was located, which, in turn, also undergoes a reorganization in its coordination sphere, shifting from a pentacoordinated to a hexacoordinated complex, ^2^ **INT2c**. This ligand reorganization process is energetically demanding, requiring 9.97 kcal/mol. Next, the O—O bond is formed through ^2^ **TS2** as a result of intramolecular oxygen coupling (IOC) due to the “scissor” vibrational motion associated with the imaginary frequency of this TS. In this vibrational mode, it was possible to observe the varying angle ∠O2–Cu–O1, which corresponds to a SET from the $\sigma_{\text{O‐O}}^{*}$ orbital to the copper (see Figure S10, Supporting Information). This O—O bond formation mechanism is kinetically feasible at room temperature and culminates in ^2^ **PC2** as the product, which was further confirmed by IRC calculations. Finally, HPO_4_^2−^ departs from ^2^ **PC1** and ^2^ **PC2**, respectively, leading to ^2^ **E** and ^2−^ **F1**. In addition, the role of proton accepting from the phosphate buffer under neutral conditions, decreasing the reaction barriers related to O—O bond formation, can be rationalized due to the prohibitive barrier (38.49 kcal/mol) of ^2^ **TS0**. The O—O bond formation by ^2−^ **TS0** was computed in the absence of a HPO_4_^2−^ anion (see details on this reaction path in Figure S11, Supporting Information).

#### 2.1.3 Catalyst Regeneration and O_2_ Release

**Figure 5:**
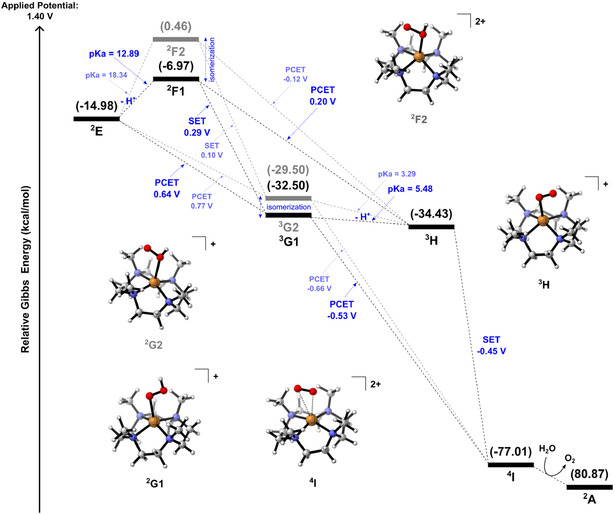
Free energy diagram of the electrochemical events leading to O_2_ release and catalyst regeneration at 298.15 K and pH = 7, with an applied potential of 1.4 V.

With the formation of ^2^ **E** and ^2^ **F1**, DFT calculations reveal a series of exergonic electrochemical events (**Figure** **5**) culminating in ^4^ **I**, in which the O_2_ is released, and the catalyst ^2^ **A** is regenerated. These electrochemical events (PCET and SET) have been described to have extremely low potentials. Specifically, the major difference between the Cu complexes ^2^ **F1** and ^2^ **F2**, as well as ^3^ **G1** and ^3^ **G2**, is whether the proton is released from oxygen atoms O1 or O2. Indeed, those coupled Cu‐containing intermediates (^2^ **F1** and ^2^ **F2**, or ^2^ **G1** and ^2^ **G2**) could be interconverted by isomerization as described in the free energy profile of the O_2_ departure (Figure 5). Spin density analysis (Figure S6, Supporting Information) reveals the copper standing in the oxidation state of +2 throughout all processes during this catalyst regeneration, so it is evident that the electron losses from these electrochemical events take place only on the oxygen atoms. The final Cu(II) intermediate ^4^ **I** exhibits the paramagnetic O_2_ with two unpaired electrons, weakly interacting with the metal center by a moderate bond length (≈2.80 Å), and thus O_2_ is ready to be released. Finally, the O_2_ is replaced by a water molecule and the cycle restarts.

### 2.2 Comparing Features from WO Catalytic Cycles: [Cu(12‐TMC)]^2+^ *vs* [Cu(14‐TMC)]^2+^

**Figure 6:**
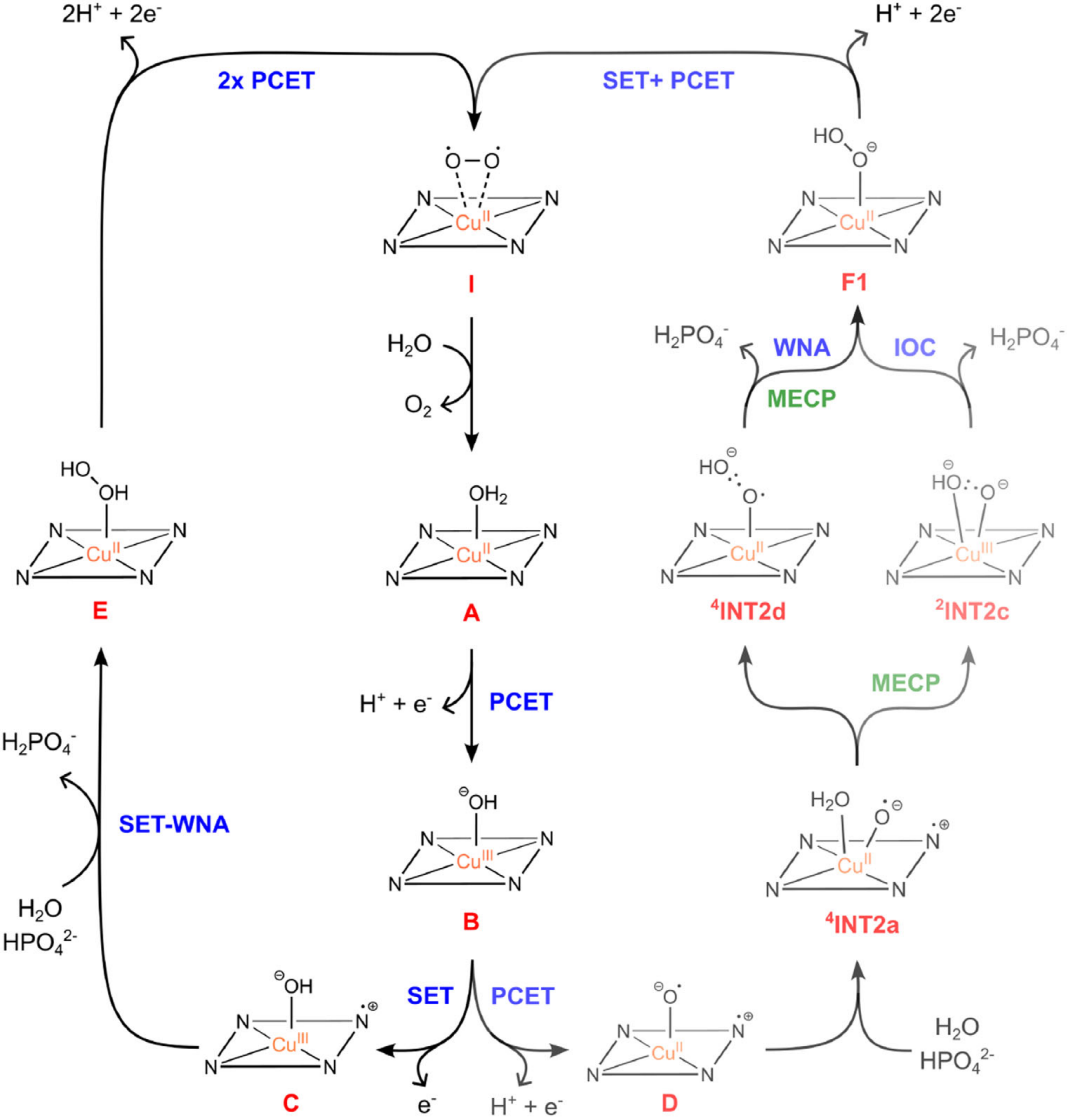
Catalyic cycle of the WO in presence of [Cu(12‐TMC)]^2+^ depicting the two main pathways through intermediates **C** (left) and **D** (right); the latter can be dismembered in two routes which leads to different O—O bond formations (WNA vs IOC mechanisms).

The WO catalytic cycle of [Cu(12‐TMC)]^2+^ based on DFT calculations is presented in **Figure** **6**. Overall, the electrochemical activation of the catalyst has similarities with the reported one for [Cu(14‐TMC)]^2+^ catalysis (see **Tables** **1** and **2**, as well as Figure S12, Supporting Information). However, there are significant features of the ligand size on these electrochemical events that are worth pointing out. The first notable difference is the inversion in stability order between ^4^ **C** and ^4^ **D**, i.e., for a larger ring ligand, 14‐TMC, the SET product is more stable than the PCET product. In addition, both structures of ^4^ **C** and ^4^ **D**, display distorted tetrahedral coordinations for [Cu(12‐TMC)]^2+^; in contrast, these geometries are not the most stable ones for [Cu(14‐TMC)]^2+^ catalysis, thus showing the size of macrocycle impacts the geometry and stability of the Cu complexes during the electrochemical activation of the catalyst.

**Table 1:** Relative Gibbs free energies (ΔG) (298.15 K) of intermediates involved in the electrochemical activation of [Cu(12‐TMC)]^2+^ and [Cu(14‐TMC)]^2+^^[^ ^37^ ^]^ catalyses.

Species	ΔG [kcal/mol]	
	**12‐TMC**	**14‐TMC**
^2^A	0.00	0.00
^3^B	2.96	2.08
^4^C	7.80	8.09
^4^D	7.04	9.47

**Table 2:** Electrochemical potentials (*E*) (298.15 K) of the [Cu(12‐TMC)]^2+^ and [Cu(14‐TMC)]^2+^^[^ ^37^ ^]^ catalyses.

Electrochemical event	*E* [V]	
	**12‐TMC**	**14‐TMC**
1st PCET	1.53	1.49
SET	1.61	1.66
2nd PCET	1.58	1.72

Regarding the O—O bond formation step, DFT calculations revealed similarities and differences comparing [Cu(12‐TMC)]^2+^ and [Cu(14‐TMC)]^2+^. It is possible to perceive that O—O bond formation mechanism from ^4^ **C** is quite similar to the mechanism reported for [Cu(14‐TMC)]^2+^ catalysis (**Table** **3**; Figure S13, Supporting Information). Furthermore, it is interesting to notice that the overall stabilization of the Cu‐containing intermediates in the doublet remains close for both catalysts. For example, the relative energies of the rate‐determining step for [Cu(14‐TMC)]^2+^ and [Cu(12‐TMC)]^2+^ catalyses were computed to be 18.96 and 19.26 kcal/mol. On the other hand, the reaction mechanism from pathway ^4^ **D** displays the most evident differences. For instance, the coordination number of [Cu(12‐TMC)]^2+^ increases, promoting a new coordination site wherein a second water molecule binds to the copper. This new rearrangement for the O—O bond formation provides a hexacoordinated (trigonal prismatic) geometry to the Cu complexes. In addition, DFT calculations show that this process is exergonic, and the mechanism follows a proton transfer from water to the HPO_4_^2−^ anion. It was also noted that, in this new coordination fashion, the doublet and quartet states on the Gibbs free energy profile are more intertwined in consequence of a pronounced multiconfigurational behavior of these Cu complexes. In this sense, two crossing points were located, revealing a more intricate mechanism for the O—O bond formation. Indeed, the IOC mechanism for O—O bond formation proposed herein by ^2^ **TS2** involving these macrocylic processes is completely new, and therefore, it was a critical feature compared with the mechanistic reports on [Cu(14‐TMC)]^2+^ catalysis.

**Table 3:** Relative Gibbs free energies (ΔG) (298.15 K) of reaction intermediates involved in the pathway ^4^C of [Cu(12‐TMC)]^2+^ and [Cu(14‐TMC)]^2+^^[^ ^3^ ^]^ catalyses.

Species	ΔG [kcal/mol]	
	**12‐TMC**	**14‐TMC**
^2^RC1	−5.19	−1.34
^2^INT1	5.47	10.02
^2^TS1	14.07	17.62
^2^PC1	−15.37	−15.35

It is convenient to point out that further discrepancies with respect to the coordination environment around the metal center have been evidenced. This can be realized through the geometry and coordination modes involving the macrocyclic complex provided by the reduction of the ligand ring size (from 14‐TMC to 12‐TMC). In other words, the manner in which the nitrogen atoms of the macrocycle are arranged around the Cu center has been directly impacted by its ligand size, as further evidenced by the bond distances and angles of the complexes. Specifically, in ^3^ **B**, for example, the σ‐donor lone pairs of the nitrogen atoms are pointed above the plane containing the four N atoms; as a result, the geometry resembles a square pyramid with the metal center above this plane (Figure S14, Supporting Information). This arrangement, provided by the reduction of the size of the macrocycle, enables the coordination of another water molecule in a distorted trigonal prismatic fashion. In addition, a recent computational study with Fe(n‐TMC) complexes (n = 12, 13, 14, 15, and 16) showed that larger macrocycles provide smaller buried volumes in the axial cavity, and hence weaker steric hindrance due to the methyl groups.^[^ ^47^ ^]^ For these reasons, when scrutinizing the [Cu(14‐TMC)OH]^2+^ structure, the four N atoms are not displayed in the same plane (Figure S14, Supporting Information), and the preferred geometry is a distorted‐trigonal bipyramidal. These preference is supported by the angles ∠N1–Cu–N3 and ∠N2–Cu–N4 computed to be 178.92° and 153.06°, respectively. As a result, the coordination of another ligand in the case of 14‐TMC would be more likely to happen on the other axial face of the macrocycle, thus leading to an octahedral geometry, which is unstable due to the imposition of considerable steric hindrance (Figure S14, Supporting Information). This implies the possibility of the coordination sphere reorganizing in different ways for 12‐TMC, producing Cu‐containing complexes in different coordination geometries

**Figure 7:**
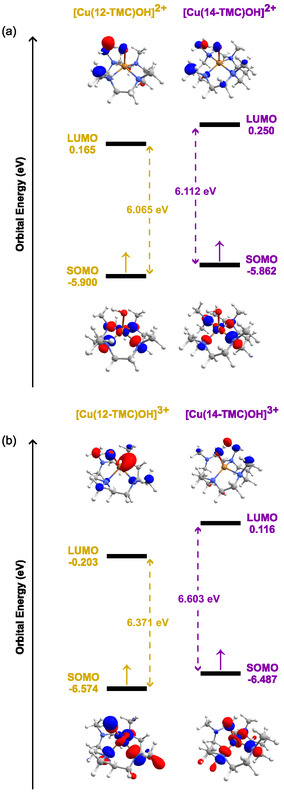
M06L/Def2‐TZVP MO diagrams of a) [Cu(12‐TMC)OH]^2+^ vs [Cu(14‐TMC)OH]^2+^ and b) [Cu(12‐TMC)OH]^3+^ vs [Cu(14‐TMC)OH]^3+^ depicting both SOMO and LUMO energies and the gaps between them.

In order to get a more detailed comparison regarding the electronic features provided by the macrocycle size reduction, we investigated the molecular orbital (MO) energy diagrams of ^3^ **B**—[Cu(12‐TMC)OH]^2+^—and its counterpart of expanded ligand size, [Cu(14‐TMC)OH]^2+^ (**Figure** **7**a). When the macrocycle shrinks (14‐TMC → 12‐TMC), the electronic energy of the Singly Occupied Molecular Orbital (SOMO), which corresponds to a *σ* ^*^ orbital with *d*_xy_ character, decreases. In this sense, the orbital analysis indicates a relatively weaker tendency for electron loss on the metal influenced by the 12‐TMC ligand, supported by SOMO stabilization; therefore, the SET becomes less favorable following the ligand ring size reduction. Moving to the Lowest Unoccupied Molecular Orbital (LUMO), which corresponds to the $\sigma_{\text{O‐H}}^{*}$ of proton abstraction, there is also an electronic difference between 14‐TMC and 12‐TMC: the variation in LUMO energy is more pronounced in 12‐TMC, thus making the SOMO–LUMO gap smaller. When analyzing the MOs of the SET product ^4^ **C** and its analogous [Cu(14‐TMC)OH]^3+^ (Figure 7b), we observe that ^4^ **C**'s LUMO becomes significantly less energetic than in [Cu(14‐TMC)OH]^3+^, making the proton abstraction (i.e., the PCET product ^4^ **D**) more viable. Hence, the MO frontier analyses corroborate the inversion of the stability discussed above regarding ^4^ **C** and ^4^ **D** in 12‐TMC.

In [Cu(12‐TMC)]^2+^ catalysis, the fact that the gaps between MOs tend to be altered also helps to explain the relative energies of critical intermediates on the O—O bond formation mechanism from pathway ^4^ **C**. Taking ^2^ **INT1** as an example, the energy gap between key orbitals involved in the SET–WNA mechanism (Figure 3a) is larger than in the analogous intermediate of [Cu(14‐TMC)]^2+^ catalysis (1.175 eV vs 0.757 eV, respectively). In this regard, considering that in order to promote the O—O bond formation, an electron is transferred from the $\sigma_{\text{O‐O}}^{*}$ orbital to an orbital of molecular (OM) with *d* character centered on the complex, the ΔG between ^2^ **INT1** and ^2^ **TS1** (Table 3; Figure S13, Supporting Information) becomes slightly lower with the expansion of the ring size (8.60 vs 7.60 kcal/mol). The larger OM gaps of 12‐TMC in this pathway also indicate a tendency of lower doublet energies, which corroborate the overall relative stabilization of these species compared with those of 14‐TMC.

## 3 Summary and Conclusions

In summary, our study presents a comprehensive overview of the mechanism of WO catalyzed by the complex [Cu(12‐TMC)]^2+^. DFT calculations were conducted to scrutinize the mechanisms of the electrochemical activation, followed by the O—O bond formation and the electrochemical events, leading to O_2_ release and the regeneration of the catalyst ^2^ **A**. In particular, for the O—O bond formation, two pathways from ^4^ **C** or ^4^ **D** as starting points derived from the electrochemical activation of the catalyst have been determined. The mechanism from ^4^ **D** demonstrates the possibility of hexacoordination and also of an IOC for O—O bond formation showing multiple MECP points, thus following two different MECP–WNA mechanisms. While for the O—O bond formation mechanism from the pathway ^4^ **C**, a SET–WNA mechanism has been proposed.

DFT calculations revealed similarities and differences comparing Cu(12‐TMC)]^2+^ and [Cu(14‐TMC)]^2+^. It is possible to perceive that O—O bond formation mechanism from ^4^ **C** follows the general trends of the mechanism reported for [Cu(14‐TMC)]^2+^ catalysis. Indeed, the relative energies of the rate‐determining step for [Cu(14‐TMC)]^2+^ and [Cu(12‐TMC)]^2+^ catalytic cycles were computed to be similar, and both follow SET–WNA mechanisms. On the other hand, the reaction pathway from ^4^ **D** displays the most evident differences in the MECP–WNA mechanism. Specifically, the IOC mechanism for O—O bond formation proposed herein involving [Cu(12‐TMC)]^2+^ was a critical feature compared with the mechanistic reports on [Cu(14‐TMC)]^2+^ catalysis. Furthermore, the reduction of the ligand size promotes the possibility of increasing the coordination number and creating new coordination modes, thus producing a more intricate mechanism for the O—O bond formation. In terms of the electronic features based on the MO frontier analyses computed from 14‐TMC to 12‐TMC, when the ligand size of the macrocycle is reduced, more feasible electrochemical events (PCET and SET) are produced. Regarding the O—O bond formation process, we noticed a possible trend that the reaction barrier of the SET–WNA mechanism turns slightly higher with the reduction of the ligand size.

In conclusion, the present work provides a comprehensive overview of the possible mechanistic routes and key intermediates that might arise from the computational ligand modelling, providing new electronic and structural findings involved in the electrochemical reaction steps of the catalytic cycles. These new computational insights can contribute to rationalize the thermodynamic and kinetic properties in terms of the macrocyclic ring size in Cu(II) complexes toward the design of novel WO electrocatalyses.

## 4 Experimental Section

4.1

4.1.1

##### Electronic Structure Methods

While [Cu(14‐TMC)]^2+^ has its X‐ray diffraction structure elucidated, we should decipher the most stable conformer for [Cu(12‐TMC)]^2+^ in aqueous solution as catalyst candidate.^[^ ^60^ ^]^ For this purpose, it was necessary to perform a wide conformational analysis; to handle this, we used the xtb‐CREST software,^[^ ^61^ ^]^ which combines metadynamical simulations based on the semiempirical quantum mechanical methods GFNn‐xTB to rapidly deliver a conformational ensemble. The complete analysis with relative energies of each conformer is depicted in Supporting Information (Section S1, Supporting Information). The energies related to these conformers were further refined using DFT calculations using the methods described below.

All stationary points, including reactants, products, the catalyst, reaction intermediates, and TSs, were obtained through DFT calculations using the Gaussian09 suite of quantum chemical programs.^[^ ^62^ ^]^ Taking into account the limited accuracy in practical electronic structure calculations based on DFT methods, especially for 3*d* transition metals and the associated catalytic systems,^[^ ^63^ ^]^ the DFT methodology employed here was previously successfully applied to [Cu(14‐TMC)]^2+^ WO catalysis as validated by benchmarking involving experimental values^[^ ^37^ ^]^ and further supported by reports in literature.^[^ ^64^, ^65^ ^]^ The density functional B3LYP^[^ ^66^ ^]^ with empirical dispersion correction D3^[^ ^67^ ^]^ was used with the Def2‐SVP basis set for all atoms,^[^ ^68^ ^]^ except for copper, for which the basis set combined with effective core potential LANL2TZ(f)^[^ ^69^, ^70^ ^]^ was applied. Single‐point calculations with a larger basis set were carried out in order to refine the electronic energies. For these calculations, we used the M06L‐D3 density functional^[^ ^71^ ^]^ with the Def2‐TZVP basis set for all atoms, except for copper, for which we kept the LANL2TZ(f).

Additionally, the minimal energy crossing points were determined using the easyMECP program,^[^ ^72^ ^]^ which uses the Harvey's algorithm to determine the critical point involving intersections between potential energy surfaces (PES) of different spin multiplicities. In the case of the TSs described herein, they were further validated (connection between a given TS and its reactant and product stationary points) by using IRC calculations.^[^ ^73^ ^]^ Spin density population analyses were carried out using the Multiwfn program.^[^ ^74^, ^75^ ^]^

##### Electrochemistry

To determine the Gibbs energies (298.15 K) in aqueous solution, the Solvent Model Density (SMD)^[^ ^76^ ^]^ continuum dielectric model was applied to all calculations (geometry optimization, frequency, and single‐point calculations). The concentration correction to the standard state of all reaction components was made (1 atm to 1 M) (except for water, the solvent, whose concentration is ≈55.56 M). Quasiharmonic free energies were finally derived (using a frequency cut‐off of 100 cm^−1^ and the rigid‐rotor‐harmonic‐oscillator approximation proposed by Grimme). These corrections were performed with the Goodvibes program.^[^ ^77^ ^]^

For electrochemical processes, the standard redox potential (E°) was evaluated using the following equation

(2) $${E{}^{\circ}}_{O|R}=\frac{G{}^{\circ}(O,\text{aq})+n^{H+}G{}^{\circ}(H^{+},\text{aq})-G{}^{\circ}(R,\text{aq})}{n_{e}F}-{E{}^{\circ}}_{\text{SHE}}$$

where G°(O,aq) and G°(R,aq) are respectively the Gibbs energies of the oxidizing and reducing agents; $G{}^{\circ}(H^{+},\text{aq})$ is the free energy of the proton in solution, given by the experimental value of −270.3 kcal/mol;^[^ ^78^ ^]^ and ${E{}^{\circ}}_{\text{SHE}}$ is the potential of the standard hydrogen electrode (SHE), 4.281 V.^[^ ^79^ ^]^ The Faraday constant F was taken as 23.08 kcal/mol. Regarding a proton‐coupled electron transfer (PCET), the value of n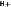 is different from zero; for SETs, on the other hand, this term vanishes.

In order to reproduce the PCET redox potential under the experimental conditions of [Cu(14‐TMC)]^2+^ catalysis (pH = 7), the Nernst equation was applied

(3) $$E_{O|R}={E{}^{\circ}}_{O|R}-\frac{n^{H+}}{n_{e}}\frac{RT}{F}\ln(10)\text{pH}$$

Considering that proton transfer processes are also recurrent in WO catalysis, we evaluated the values of *pK_a_* through

(4) $$pK_{a}=\frac{G{}^{\circ}(A^{-},\text{aq})+n^{H+}G{}^{\circ}(H^{+},\text{aq})-G{}^{\circ}(\text{AH},\text{aq})}{RT\ln(10)}$$

where $A^{-}$ and AH are, respectively, the base and acid.

## Conflict of Interest

The authors declare no conflict of interest.

## Supporting information

Supplementary Material

## Data Availability

The data that support the findings of this study are available from the corresponding author upon reasonable request.
