# Supplementary material for: Electrochemical Water Oxidation Mechanisms Involving Macrocyclic Copper(II) Complexes: Ligand Ring Size Effects on Catalytic Cycles
Source: Chemphyschem. 2025 Dec 10;27(4):e202500637. doi: 10.1002/cphc.202500637 (PMC12931582; doi:10.1002/cphc.202500637)
Supplement: Supplementary file 1 — Supplementary Material [file CPHC-27-e202500637-s001.pdf]

Supporting Information

Electrochemical Water Oxidation Mechanisms involving Macrocyclic Copper(II) Complexes: Ligand Ring Size Effects on Catalytic Cycles

João Pedro C. S. Neves†, Roberto Rivelino, Tiago Vinicius Alves*,† and Vitor H. Menezes da Silva*,†

†*Departamento de Físico-Química, Instituto de Química, Universidade Federal da Bahia, Rua Barão de Jeremoabo, 147, Salvador, Bahia, 40170-115, Brazil*
‡*Instituto de Física, Universidade Federal da Bahia, Salvador, Bahia 40210-340, Brazil*

*E-mail address: tiagova@ufba.br, vhugomenezes@gmail.com or vhugo@iq.usp.br

Sections

S1. Conformational Analysis 4

S2. Spin Densities 5

S3. Alternative Structures of Selected Intermediates 7

S4. Additional Information of Pathway ^{4}**D** 8

S5. Additional Pathways for O-O Bond Formation 10

S6. Additional Information on 12-TMC vs 14-TMC 11

S7. Additional Information of Pathway ^{4}C 13

S8. Cartesian Coordinates 14

Tables

S1. Charge transfer analysis of ^{4}**INT2d** and ^{2}**PC2** employing Voronoi[1] and Hirshfeld[2] calculations 9

Figures

S1. Esteroisomers of $[Cu(12\text{-}TMC)]^{2+}$ 4

S2. Relative energies between the conformers of $[Cu(12\text{-}TMC)]^{2+}$. 4

S3. Spin densities of the electrochemical activation. 5

S4. Spin densities of the pathway ^{4}**C**. 5

S5. Spin densities of the pathway ^{4}**D**. 6

S6. Spin densities of the O_2 release and catalyst recovering. 6

S7. Alternative structures of **C** and **D** with respective spin densities; H atoms attached to carbon were omitted for clarity. 7

S8. Minimal energy crossing point structures displayed in Figure 4. 8

S9. Mechanism of the OH^- decoordination in quartet route of Pathway ^{4}**D**; H atoms attached to carbon were omitted for clarity. 8

S10. Mechanism of the O-O bond formation in doublet route of Pathway ^{4}**D**; H atoms attached to carbon were omitted for clarity. 9
S11. Gibbs energy profile of the Pathway ^{4}**D** without the phosphate buffer. . . . 10
S12. Comparison of electrochemical activation PES between 12-TMC (red) and 14-TMC [3] (blue) catalyses. 11
S13. Comparison of Pathway ^{4}**C**'s PES between 12-TMC (red) and 14-TMC [3] (blue) catalyses. 11
S14. Geometries of selected intermediates of $[Cu(12\text{-}TMC)]^{2+}$ and $[Cu(14\text{-}TMC)]^{2+}$ catalyses; for better visualization, C atoms were omitted as well as hydrogens attached to them. 12
S15. IRC of ^{2}**TS1** depicting an analogous geometry of ^{2}**INT1**. 13

S1. Conformational Analysis

Initial guess structures of the conformers in Figure S2 were obtained through calculations with CREST software, which in turn were submitted to geometry optimizations with B3LYP-D3/Def2-SVP. Final energies took into account single point calculations with M06L-D3/Def2-TZVP. The main difference between conformers of each stereoisomer (Figure S1) arise from the possibility of torsion in C-C σ bonds of the side chains between nitrogens, and their multiple combinations.

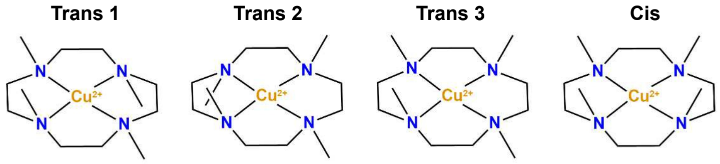

Fig. S1.: Esteroisomers of $[Cu(12\text{-TMC})]^{2+}$

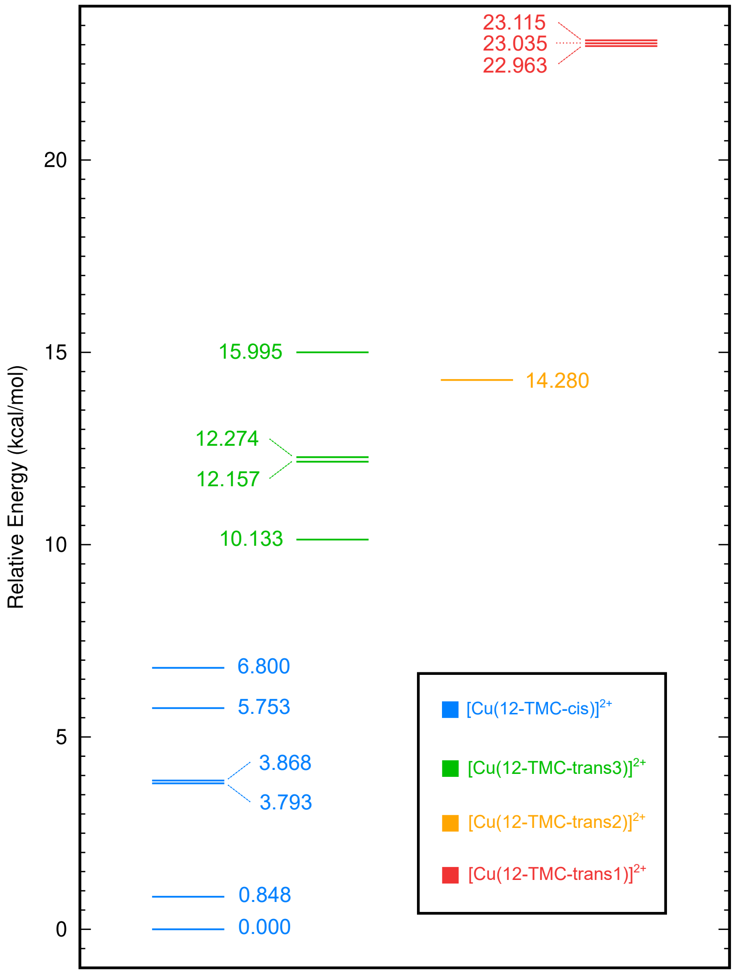

Fig. S2.: Relative energies between the conformers of $[Cu(12\text{-TMC})]^{2+}$.

S2. Spin Densities

Spin Densities (ρ) - Electrochemical Activation

^{2}**A**	^{3}**B**	^{4}**C** (η^3)	^{4}**D** (η^3)
ρ_{Cu} = 0.581	ρ_{Cu} = 0.913	ρ_{Cu} = 0.834	ρ_{Cu} = 0.726
ρ_{L} = 0.419	ρ_{L} = 0.723	ρ_{L} = 0.696 (N4) / 0.941	ρ_{L} = 0.529 (N4) / 0.655
ρ_{O} < 0.001	ρ_{O} = 0.364	ρ_{O} = 0.411	ρ_{O} = 1.090

Fig. S3.: Spin densities of the electrochemical activation.

Spin Densities (ρ) - O-O Bond Formation Pathway C

^{2}**RC1**	^{2}**INT1**	^{2}**TS1**	^{2}**PC1**
ρ_{Cu} = 0.012	ρ_{Cu} = 0.667	ρ_{Cu} = 0.446	ρ_{Cu} = 0.575
ρ_{L} = 0.644 (N4) \ 0.257	ρ_{L} = -0.652 (N4) \ -0.075	ρ_{L} = 0.567 (N4) \ 0.501	ρ_{L} = 0.425
ρ_{O1} = 0.014	ρ_{O1} = 0.749	ρ_{O1} = -0.138	ρ_{O1} < 0.001
ρ_{O2} = 0.015	ρ_{O2} = 0.209	ρ_{O2} = -0.392	ρ_{O2} < 0.001
ρ_{Phos} = 0.038	ρ_{Phos} = -0.048	ρ_{Phos} = 0.016	ρ_{Phos} < 0.001

Fig. S4.: Spin densities of the pathway ^{4}**C**.

Spin Densities (ρ) - O-O Bond Formation Pathway D

^{4}RC2	^{4}INT2a	^{4}INT2b	^{2}INT2b	^{4}INT2c
ρ_{Cu} = 0.726	ρ_{Cu} = 0.674	ρ_{Cu} = 0.667	ρ_{Cu} = -0.009	ρ_{Cu} = 0.798
ρ_{L} = 0.565 (N4) \ 0.446	ρ_{L} = 0.662 (N4) \ 0.522	ρ_{L} = 0.567 (N4) \ 0.018	ρ_{L} = 0.136 (N4) \ 0.015	ρ_{L} = 0.748
ρ_{O1} = 0.997	ρ_{O1} = 1.039	ρ_{O1} = 1.110	ρ_{O1} = 0.791	ρ_{O1} = 1.115
ρ_{O2} = 0.016	ρ_{O2} = 0.101	ρ_{O2} = 0.153	ρ_{O2} = 0.059	ρ_{O2} = 0.328
ρ_{Phos} = 0.250	ρ_{Phos} = 0.002	ρ_{Phos} = 0.004	ρ_{Phos} = 0.008	ρ_{Phos} = 0.011

^{2}INT2c	^{4}TS2	^{4}INT2d	^{2}TS2	^{2}PC2
ρ_{Cu} = 0.015	ρ_{Cu} = 0.772	ρ_{Cu} = 0.672	ρ_{Cu} = 0.748	ρ_{Cu} = 0.590
ρ_{L} = -0.097	ρ_{L} = 0.644	ρ_{L} = 0.497	ρ_{L} = 0.672	ρ_{L} = 0.411
ρ_{O1} = 0.957	ρ_{O1} = 1.097	ρ_{O1} = 1.281	ρ_{O1} = -0.342	ρ_{O1} < 0.001
ρ_{O2} = 0.139	ρ_{O2} = 0.494	ρ_{O2} = 0.551	ρ_{O2} = -0.097	ρ_{O2} = -0.001
ρ_{Phos} = -0.014	ρ_{Phos} = -0.007	ρ_{Phos} = -0.001	ρ_{Phos} = 0.019	ρ_{Phos} < 0.001

Fig. S5.: Spin densities of the pathway ^{4}**D**.

Spin Densities (ρ) - O_2 Release / Catalyst Regeneration

^{2}E	^{2}F1	^{2}F2	^{3}G1	^{3}G2
ρ_{Cu} = 0.573	ρ_{Cu} = 0.597	ρ_{Cu} = 0.581	ρ_{Cu} = 0.586	ρ_{Cu} = 0.593
ρ_{L} = 0.427	ρ_{L} = 0.402	ρ_{L} = 0.417	ρ_{L} = 0.435	ρ_{L} = 0.448
ρ_{O1} < 0.001	ρ_{O1} = 0.001	ρ_{O1} < 0.001	ρ_{O1} = 0.637	ρ_{O1} = 0.298
ρ_{O2} < 0.001	ρ_{O2} < 0.001	ρ_{O2} = 0.002	ρ_{O2} = 0.342	ρ_{O2} = 0.661

^{3}H		^{4}I	
ρ_{Cu} = 0.580	ρ_{O1} = 0.466	ρ_{Cu} = 0.592	ρ_{O1} = 0.962
ρ_{L} = 0.365	ρ_{O2} = 0.589	ρ_{L} = 0.450	ρ_{O2} = 0.996

Fig. S6.: Spin densities of the O_2 release and catalyst recovering.

S3. Alternative Structures of Selected Intermediates

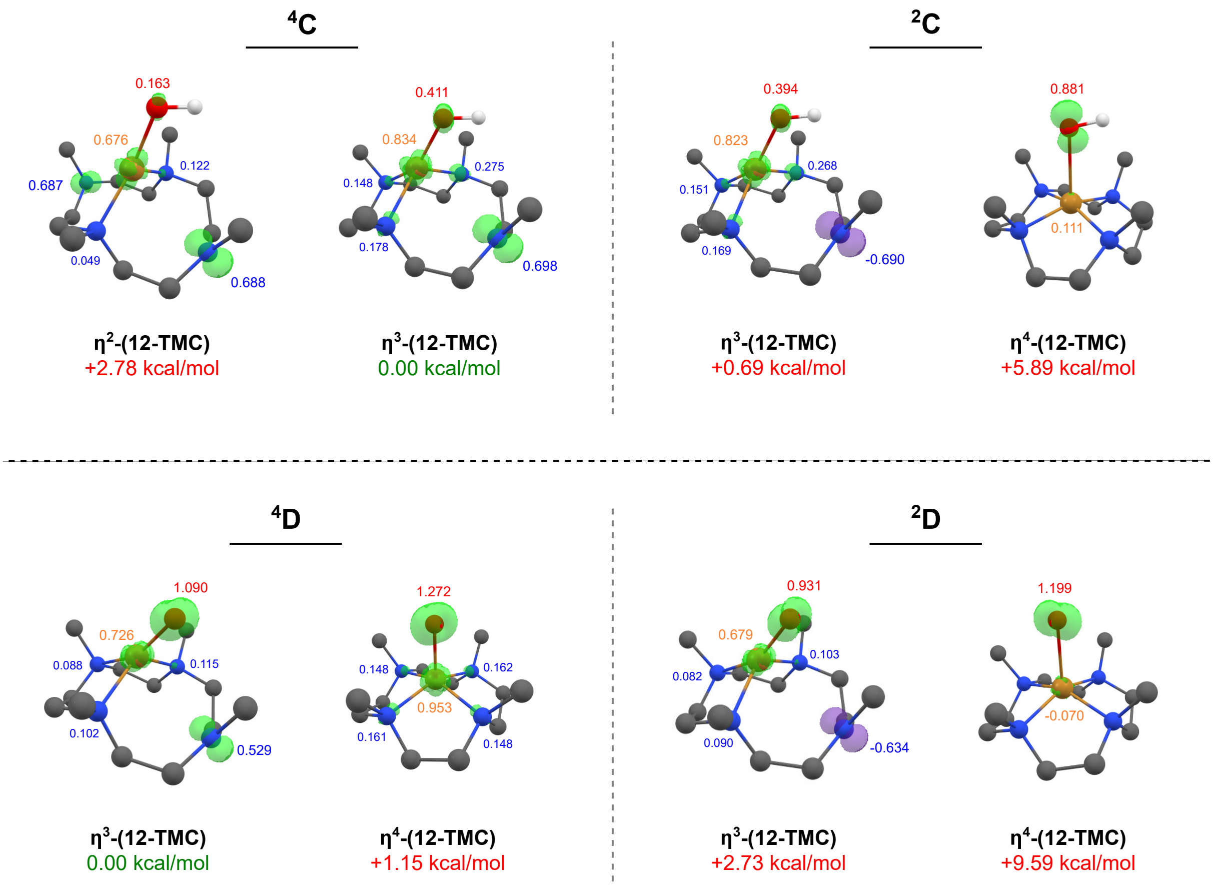

Fig. S7.: Alternative structures of **C** and **D** with respective spin densities; H atoms attached to carbon were omitted for clarity.

S4. Additional Information of Pathway ^{4}D

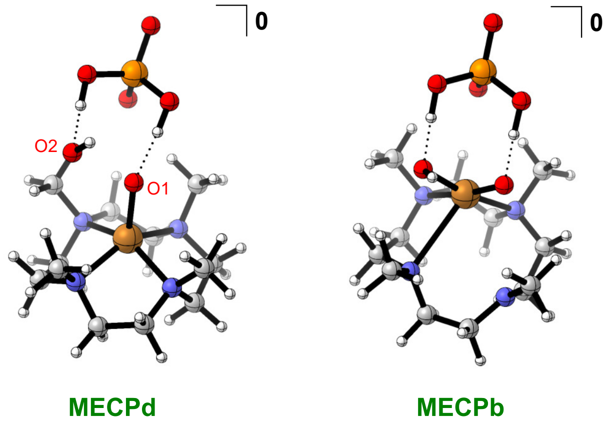

Fig. S8.: Minimal energy crossing point structures displayed in Figure 4.

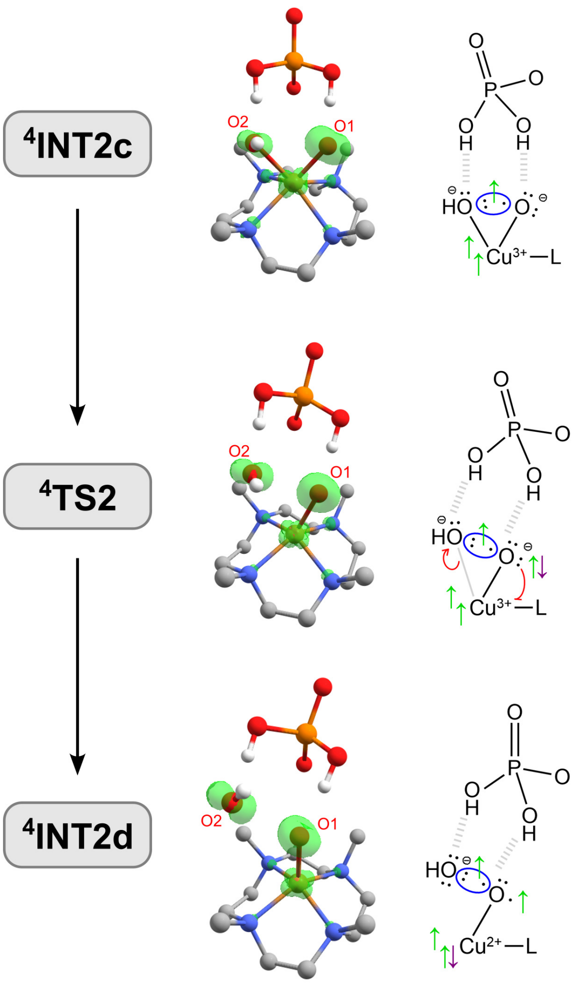

Fig. S9.: Mechanism of the OH^- decoordination in quartet route of Pathway ^{4}**D**; H atoms attached to carbon were omitted for clarity.

Table S1.: Charge transfer analysis of ^{4}**INT2d** and ^{2}**PC2** employing Voronoi[1] and Hirshfeld[2] calculations

System	Atom	Voronoi	Hirshfeld
^{4}INT2d	O1	-0.240	-0.188
	O2	-0.335	-0.324
^{2}PC2	O1	-0.358	-0.297
	O2	-0.163	-0.132

[1] Fonseca Guerra, C.; Handgraaf, J.-W.; Baerends, E. J.; Bickelhaupt, F. M. Voronoi deformation density (VDD) charges: Assessment of the Mulliken, Bader, Hirshfeld, Weinhold, and VDD methods for charge analysis. J. Comput. Chem. 2004, 25, 189–210 DOI: 10.1002/jcc.10351

[2] Hirshfeld, F. L. Bonded-Atom Fragments for Describing Molecular Charge Densities. Theor. Chim. Acta 1977, 44 (2), 129–138, DOI: 10.1007/BF00549096

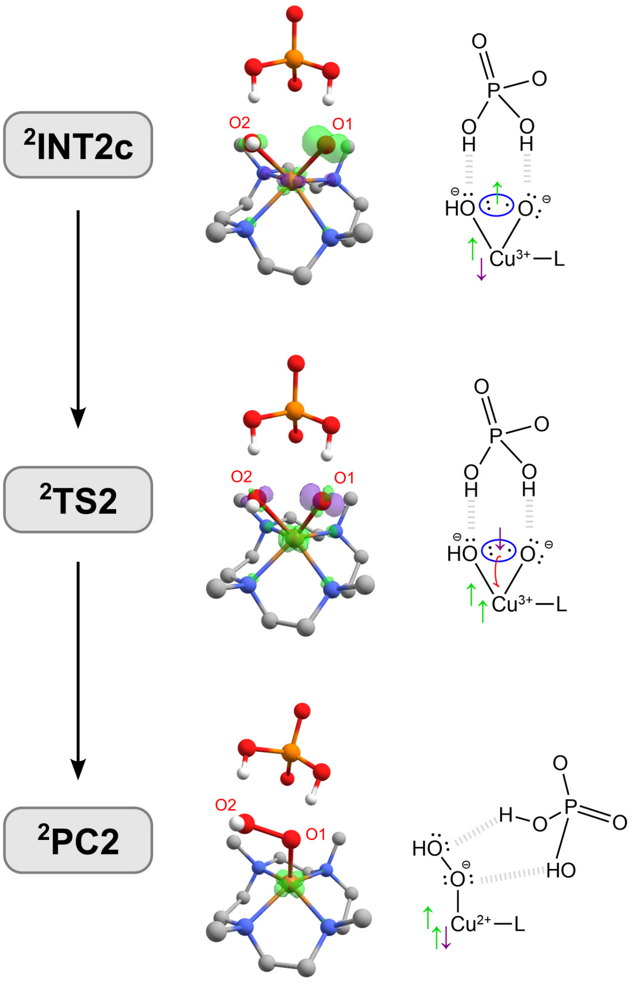

Fig. S10.: Mechanism of the O-O bond formation in doublet route of Pathway ^{4}**D**; H atoms attached to carbon were omitted for clarity.

S5. Additional Pathways for O-O Bond Formation

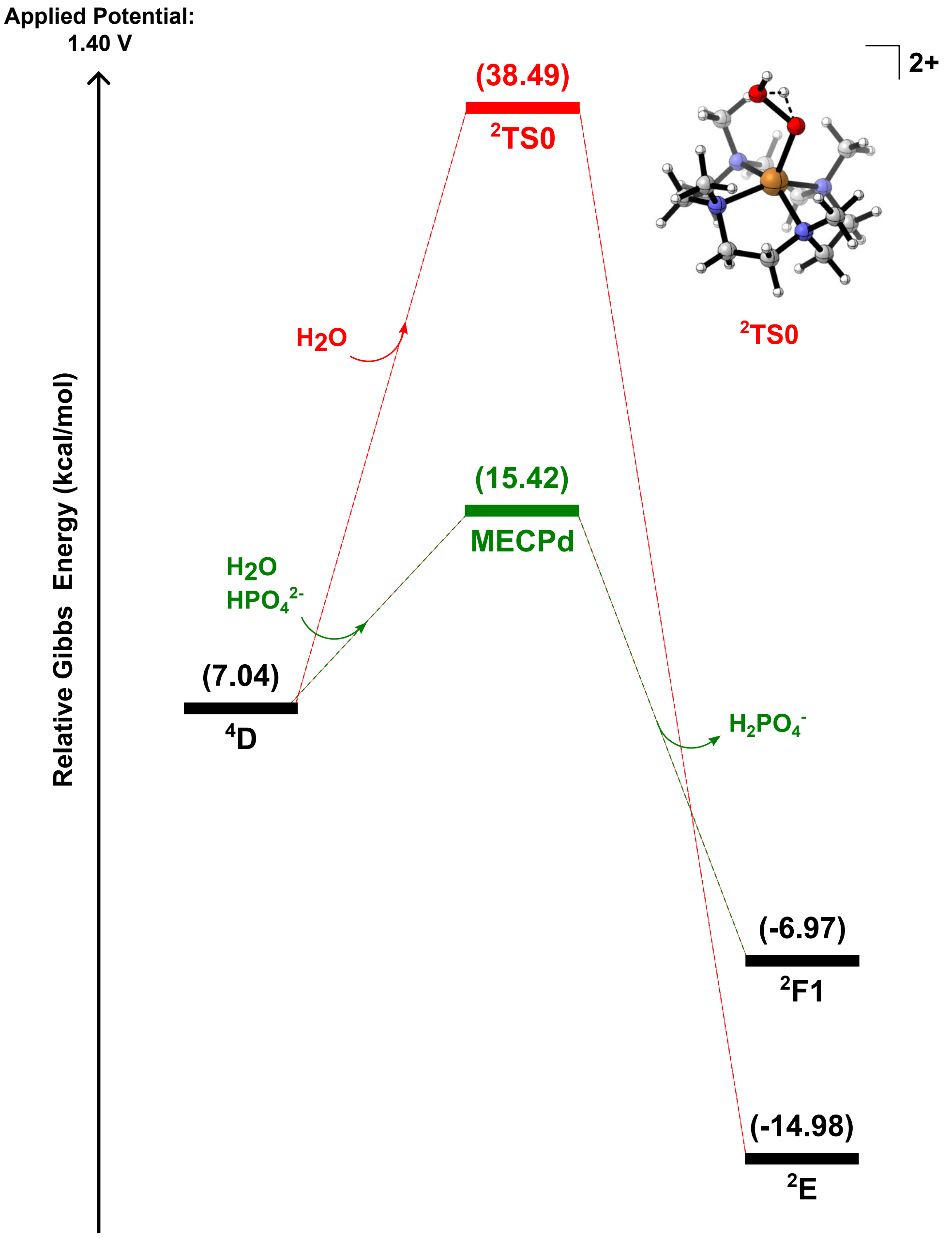

Fig. S11.: Gibbs energy profile of the Pathway ^{4}**D** without the phosphate buffer.

S6. Additional Information on 12-TMC vs 14-TMC

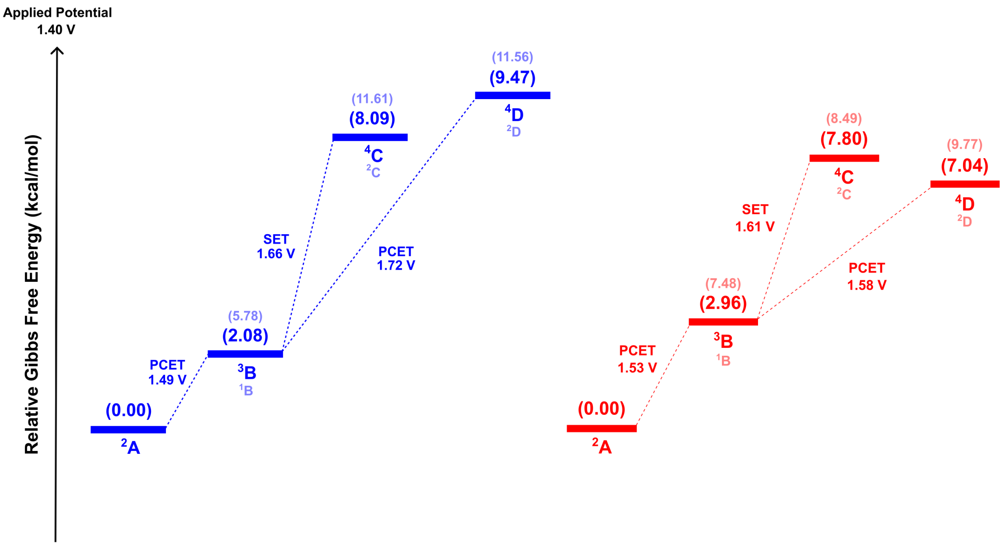

Fig. S12.: Comparison of electrochemical activation PES between 12-TMC (red) and 14-TMC [3] (blue) catalyses.

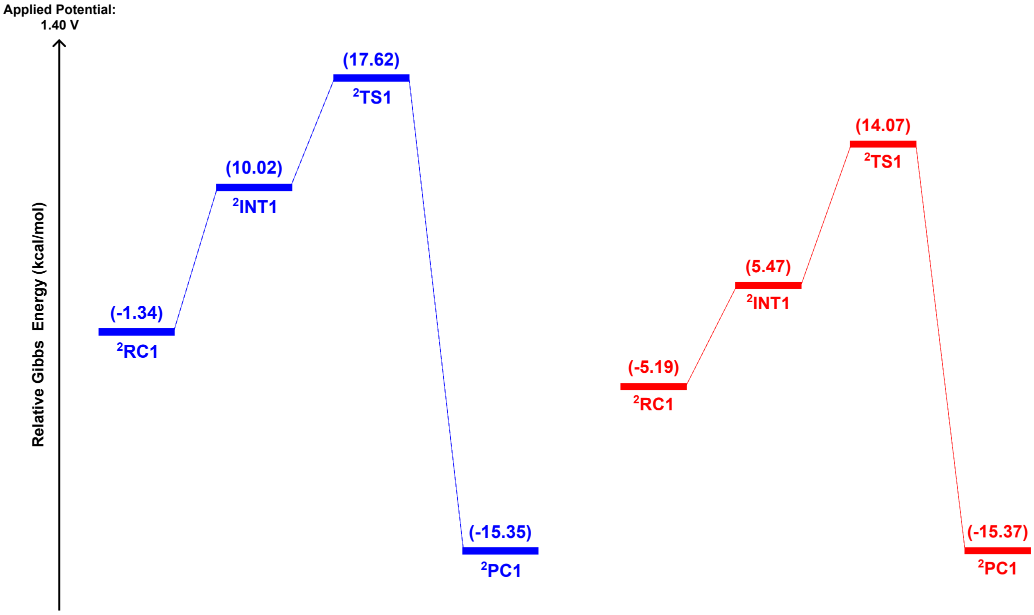

Fig. S13.: Comparison of Pathway ^{4}**C**'s PES between 12-TMC (red) and 14-TMC [3] (blue) catalyses.

[3] Neves, J. P. C. S.; Nascimento, J. L.; Sampaio, B. S.; Rivelino, R.; Alves, T. V.; Da Silva, V. H. M. Theoretical Screening of the Water Oxidation Electrocatalytic Cycle Promoted by Single-Site Macrocyclic Copper(II) Complexes: Unraveling the Role of the HPO_4^{2-} Anion under Neutral Conditions. ACS Org. Inorg. Au, 2025, XXXX(XXX), XXX-XXX, DOI: 10.1021/acsorginorgau.5c00047

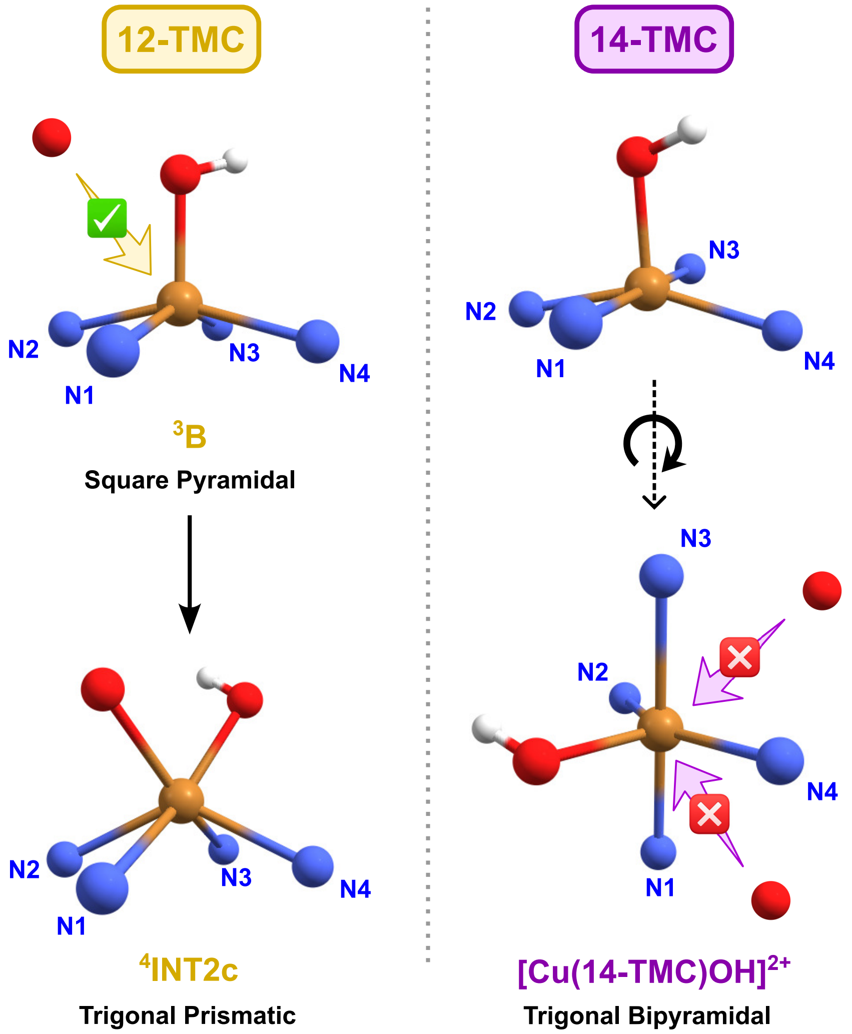

Fig. S14.: Geometries of selected intermediates of [Cu(12-TMC)]$^{2+}$ and [Cu(14-TMC)]$^{2+}$ catalyses; for better visualization, C atoms were omitted as well as hydrogens attached to them.

Unlike in 14-TMC, the conformation of 12-TMC ligand facilitates the hexacoordinated structure through a square pyramidal geometry. In the case of 14-TMC, the coordination in equatorial positions is hampered by steric hindrance and torsional strain in the macrocycle.

S7. Additional Information of Pathway ^{4}C

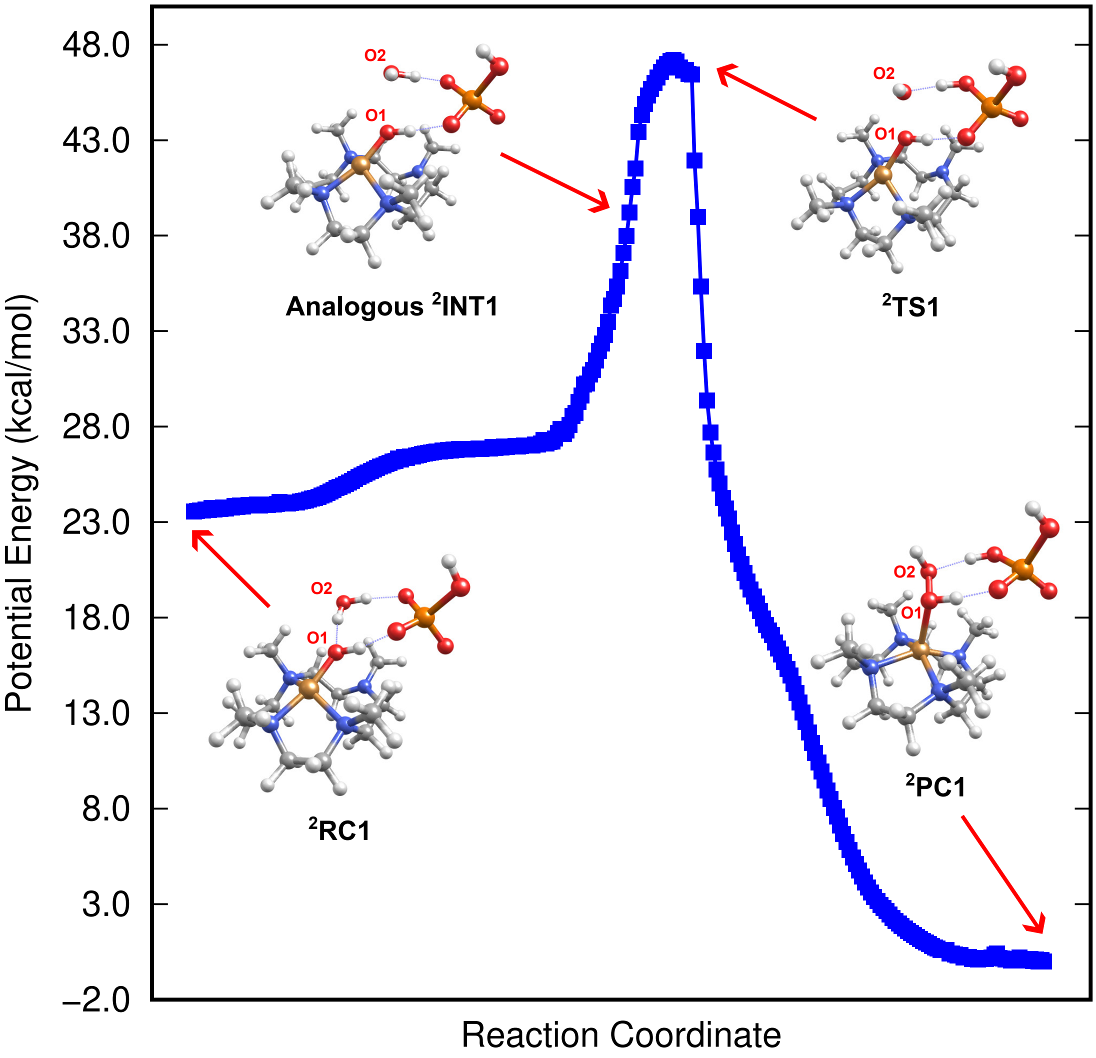

Fig. S15.: IRC of ^{2}**TS1** depicting an analogous geometry of ^{2}**INT1**.

S8. Cartesian Coordinates

^{2}A

E(UM06L) = -965.724622596

29	-0.962613000	-0.916914000	2.171014000
6	-3.448254000	-2.434524000	2.795682000
1	-2.775814000	-3.175351000	3.245384000
1	-3.687729000	-1.684400000	3.560228000
1	-4.377627000	-2.938634000	2.475809000
6	-3.631639000	-0.678351000	1.101945000
6	-2.771676000	0.477565000	0.606494000
1	-2.187903000	0.162095000	-0.269677000
6	-2.475119000	1.571256000	2.791895000
1	-3.201119000	0.891677000	3.254923000
1	-1.720422000	1.831100000	3.546025000
1	-2.995563000	2.491334000	2.471040000
6	1.508922000	0.598286000	2.811175000
1	1.721155000	-0.149366000	3.586642000
1	2.450920000	1.089625000	2.508836000
1	0.837659000	1.349990000	3.244010000
6	1.712787000	-1.155757000	1.117291000
1	2.353809000	-0.782962000	0.301325000
1	2.382978000	-1.494764000	1.916741000
6	0.858303000	-2.315158000	0.619537000
1	1.504147000	-3.157702000	0.314562000
1	0.279707000	-2.003139000	-0.261248000
7	-0.106042000	-2.749727000	1.661918000
7	-2.790608000	-1.770462000	1.658423000
7	-1.813073000	0.914540000	1.653112000
7	0.866554000	-0.062807000	1.663706000
1	-4.307174000	-0.334940000	1.895015000
1	-4.266777000	-1.053349000	0.282336000
1	-3.414092000	1.320217000	0.294565000
6	0.549704000	-3.413822000	2.800000000
1	-0.200181000	-3.630972000	3.572004000
1	1.029936000	-4.357286000	2.484580000
1	1.312929000	-2.758608000	3.237838000
6	-0.721064000	1.754473000	1.094264000
6	0.440239000	0.894059000	0.611555000
1	0.132861000	0.308573000	-0.266368000
1	-0.383591000	2.438388000	1.882550000
1	-1.093804000	2.381010000	0.267130000
1	1.285692000	1.536043000	0.306397000
1	-2.039241000	-2.142223000	-0.266085000
1	-3.197663000	-3.368802000	0.297158000
6	-2.354592000	-2.727096000	0.609344000
6	-1.198403000	-3.588102000	1.101686000
1	-1.541876000	-4.266433000	1.892308000
1	-0.823102000	-4.220516000	0.280159000
8	-0.921733000	-0.891191000	4.439414000
1	-0.766850000	0.040490000	4.664631000
1	-0.074674000	-1.316886000	4.648937000

^{3}B

E(UM06L) = -965.053246624

29	-0.957679000	-0.919994000	2.150835000
6	-3.456058000	-2.428200000	2.809954000
1	-2.789940000	-3.175686000	3.255934000
1	-3.683268000	-1.671917000	3.571085000
1	-4.390587000	-2.918990000	2.487348000
6	-3.635343000	-0.676695000	1.103043000
6	-2.769576000	0.476248000	0.612372000
1	-2.178680000	0.160174000	-0.258682000
6	-2.485208000	1.556660000	2.816834000
1	-3.232441000	0.881168000	3.249326000
1	-1.736000000	1.777490000	3.586572000
1	-2.979130000	2.492053000	2.501786000
6	1.533413000	0.606530000	2.823948000
1	1.779728000	-0.144707000	3.584267000
1	2.458716000	1.109576000	2.494322000
1	0.858769000	1.344930000	3.271958000
6	1.718285000	-1.160457000	1.131179000
1	2.348478000	-0.785429000	0.308821000
1	2.392465000	-1.499224000	1.926116000
6	0.856498000	-2.314418000	0.633116000
1	1.497363000	-3.157630000	0.323782000
1	0.272037000	-2.000434000	-0.242846000
7	-0.098581000	-2.763008000	1.681384000
7	-2.802658000	-1.775858000	1.664917000
7	-1.821106000	0.919645000	1.669663000
7	0.884320000	-0.056491000	1.681079000
1	-4.317555000	-0.336029000	1.890442000
1	-4.258174000	-1.054765000	0.276295000
1	-3.405054000	1.321528000	0.296996000
6	0.557772000	-3.416053000	2.825552000
1	-0.192590000	-3.626802000	3.597275000
1	1.032291000	-4.359186000	2.504562000
1	1.322790000	-2.758110000	3.254943000
6	-0.717655000	1.757981000	1.127066000
6	0.435451000	0.896150000	0.629691000
1	0.120938000	0.309262000	-0.244489000
1	-0.379648000	2.427708000	1.926356000
1	-1.088869000	2.393416000	0.306867000
1	1.280387000	1.533848000	0.318304000
1	-2.030380000	-2.153237000	-0.254228000
1	-3.192745000	-3.375827000	0.307165000
6	-2.351240000	-2.734789000	0.621328000
6	-1.202598000	-3.596321000	1.129533000
1	-1.547784000	-4.263570000	1.927642000
1	-0.823589000	-4.233672000	0.314455000
8	-0.974588000	-0.913246000	4.053673000
1	-0.105845000	-1.223632000	4.364040000

^{4}C

E(UM06L) = -964.836292681

29	-0.297786000	-1.172668000	2.542698000
6	-3.581471000	-2.862698000	2.991237000
1	-3.328895000	-3.920722000	3.114701000
1	-3.060311000	-2.252560000	3.751114000
1	-4.663642000	-2.700823000	3.133243000
6	-3.675399000	-1.074501000	1.270067000
6	-2.587161000	-0.085282000	0.857533000
1	-2.010914000	-0.480274000	0.013851000
6	-2.294704000	0.819396000	3.147711000
1	-2.895424000	0.023371000	3.598134000
1	-1.516725000	1.115793000	3.862093000
1	-2.939172000	1.687408000	2.924010000
6	2.086808000	0.246923000	2.114824000
1	2.483034000	-0.569816000	2.733206000
1	2.902114000	0.682415000	1.512936000
1	1.667282000	1.019233000	2.772299000
6	1.544271000	-1.347888000	0.321317000
1	1.843687000	-0.906279000	-0.641320000
1	2.443668000	-1.787291000	0.767260000
6	0.471326000	-2.396144000	0.095926000
1	0.855154000	-3.213477000	-0.538665000
1	-0.395353000	-1.953054000	-0.407396000
7	0.022058000	-2.969429000	1.391042000
7	-3.209706000	-2.383643000	1.687640000
7	-1.639221000	0.349275000	1.908662000
7	1.031742000	-0.285778000	1.229565000
1	-4.327053000	-0.671600000	2.052839000
1	-4.292865000	-1.235190000	0.366977000
1	-3.127553000	0.806021000	0.493148000
6	1.101503000	-3.700341000	2.096701000
1	0.696414000	-4.163682000	3.001642000
1	1.505793000	-4.478051000	1.427198000
1	1.904570000	-3.013962000	2.386045000
6	-0.728347000	1.403642000	1.377191000
6	0.320344000	0.787111000	0.483523000
1	-0.133635000	0.348495000	-0.413391000
1	-0.258246000	1.892978000	2.239311000
1	-1.313045000	2.165705000	0.836530000
1	1.042930000	1.547360000	0.147028000
1	-2.341513000	-2.691163000	-0.185150000
1	-3.142321000	-4.085409000	0.556963000
6	-2.472953000	-3.227811000	0.759779000
6	-1.174981000	-3.840510000	1.285612000
1	-1.339953000	-4.295466000	2.269624000
1	-0.916544000	-4.659692000	0.592598000
8	-0.485562000	-2.050287000	4.122612000
1	-0.933306000	-2.913141000	4.131152000

^{4}D

E(UM06L) = -964.377136229

29	-0.933604000	-1.330136000	2.401108000
6	-4.136351000	-2.726744000	1.972440000
1	-4.294637000	-3.814054000	1.998392000
1	-3.390215000	-2.470660000	2.810026000
1	-5.060156000	-2.170898000	2.177627000
6	-3.759168000	-0.963095000	0.263038000
6	-2.538930000	-0.037775000	0.242811000
1	-1.758101000	-0.484276000	-0.383193000
6	-2.916488000	0.917700000	2.478781000
1	-3.666092000	0.164783000	2.750185000
1	-2.395093000	1.223662000	3.395128000
1	-3.422779000	1.795273000	2.037873000
6	1.590529000	-0.133253000	2.746089000
1	1.747420000	-0.979001000	3.429037000
1	2.567822000	0.260360000	2.417650000
1	1.050561000	0.653834000	3.288588000
6	1.464336000	-1.682214000	0.848222000
1	2.047653000	-1.281248000	0.004151000
1	2.177854000	-2.162380000	1.528815000
6	0.443168000	-2.689594000	0.341902000
1	0.955860000	-3.552107000	-0.119953000
1	-0.178621000	-2.218250000	-0.429546000
7	-0.453086000	-3.147539000	1.435937000
7	-3.543121000	-2.313823000	0.759660000
7	-1.935862000	0.340805000	1.543298000
7	0.787997000	-0.582735000	1.588817000
1	-4.591109000	-0.523171000	0.823594000
1	-4.079508000	-1.054800000	-0.789343000
1	-2.882256000	0.879568000	-0.270359000
6	0.268597000	-3.958873000	2.440582000
1	-0.429974000	-4.240441000	3.238872000
1	0.691722000	-4.871016000	1.983359000
1	1.077147000	-3.371608000	2.891988000
6	-0.807154000	1.280432000	1.301894000
6	0.397969000	0.556413000	0.718412000
1	0.172642000	0.164541000	-0.281464000
1	-0.544876000	1.728536000	2.269398000
1	-1.120120000	2.099625000	0.631215000
1	1.241559000	1.258093000	0.607848000
1	-2.171884000	-2.661162000	-0.774737000
1	-3.260116000	-3.998667000	-0.397630000
6	-2.632768000	-3.209286000	0.053959000
6	-1.595513000	-3.938181000	0.916773000
1	-2.091514000	-4.405276000	1.776416000
1	-1.199909000	-4.759924000	0.293601000
8	-2.049193000	-1.929214000	3.747044000

^{2}RC1

E(UM06L) = -1684.73083445

29	-0.088623000	-1.904217000	2.419832000
6	-3.750738000	-4.142591000	2.944286000
1	-4.069005000	-4.997378000	2.330899000
1	-3.008312000	-4.505213000	3.681313000
1	-4.582922000	-3.701988000	3.506750000
6	-3.347269000	-1.761846000	2.482024000
6	-2.596769000	-0.651309000	1.763531000
1	-2.446237000	-0.890427000	0.704497000
6	-1.423132000	0.453804000	3.644658000
1	-1.963530000	-0.188324000	4.348192000
1	-0.422063000	0.663925000	4.042007000
1	-1.973710000	1.399938000	3.508773000
6	2.136866000	-0.416726000	1.900016000
1	2.663707000	-1.186801000	2.477741000
1	2.844670000	0.091690000	1.224803000
1	1.705478000	0.313050000	2.594866000
6	1.654994000	-2.138384000	0.221689000
1	1.873684000	-1.721172000	-0.772859000
1	2.605802000	-2.456111000	0.664756000
6	0.682887000	-3.290054000	0.125663000
1	1.139240000	-4.162310000	-0.369223000
1	-0.195924000	-2.997242000	-0.460203000
7	0.215390000	-3.685187000	1.495115000
7	-3.117405000	-3.149363000	2.113906000
7	-1.277199000	-0.217296000	2.335460000
7	1.075585000	-1.070287000	1.097546000
1	-3.219446000	-1.709802000	3.586157000
1	-4.431079000	-1.595131000	2.334225000
1	-3.248073000	0.237122000	1.793116000
6	1.233891000	-4.437711000	2.254404000
1	0.863365000	-4.609081000	3.270510000
1	1.426217000	-5.404289000	1.760476000
1	2.163266000	-3.858288000	2.303966000
6	-0.592523000	0.694071000	1.356922000
6	0.208307000	-0.110016000	0.357615000
1	-0.445217000	-0.694434000	-0.303315000
1	0.055972000	1.369234000	1.925853000
1	-1.343128000	1.317649000	0.847420000
1	0.833400000	0.545836000	-0.269628000
1	-2.012774000	-2.782718000	0.374940000
1	-2.946218000	-4.287346000	0.411582000
6	-2.297365000	-3.627826000	1.008668000
6	-1.058485000	-4.452014000	1.426612000
1	-1.216231000	-4.934092000	2.396463000
1	-0.912703000	-5.247249000	0.678007000
8	-0.723439000	-2.716467000	3.886808000
8	-1.228770000	-5.182411000	4.864071000
1	-0.959619000	-4.334685000	4.440728000
1	-4.224841000	-1.360831000	7.751840000
8	-3.728167000	-2.193898000	7.743663000
15	-3.673611000	-2.785635000	6.179986000
8	-5.070185000	-2.593133000	5.574764000
8	-3.210229000	-4.242496000	6.373105000
8	-2.592054000	-1.905046000	5.453218000
1	-1.481083000	-2.331959000	4.439285000
1	-1.958929000	-4.871226000	5.470315000

^{2}INT1

E(UM06L) = -1684.70591270

29	-1.793842000	-1.213457000	2.658986000
6	-4.113015000	-2.721150000	2.061812000
1	-3.578028000	-3.535103000	2.565941000
1	-4.603185000	-2.109219000	2.830401000
1	-4.877549000	-3.149978000	1.391076000
6	-3.862395000	-0.697191000	0.720110000
6	-2.905115000	0.473114000	0.601377000
1	-2.154181000	0.263786000	-0.170518000
6	-3.085762000	1.405249000	2.859226000
1	-3.982417000	0.799202000	3.040567000
1	-2.549089000	1.525304000	3.805565000
1	-3.390636000	2.395293000	2.476421000
6	1.866111000	1.027973000	2.780191000
1	1.294440000	1.174490000	3.713104000
1	2.829929000	0.591698000	3.069940000
1	1.989170000	1.995643000	2.276951000
6	1.424654000	-1.297184000	2.133132000
1	2.502529000	-1.412006000	1.914566000
1	1.364420000	-1.471096000	3.233135000
6	0.642711000	-2.335626000	1.343157000
1	1.323527000	-3.188281000	1.177594000
1	0.383729000	-1.956715000	0.347161000
7	-0.590017000	-2.858637000	1.996547000
7	-3.169390000	-1.871758000	1.304578000
7	-2.205498000	0.716375000	1.891691000
7	1.137748000	0.112313000	1.935763000
1	-4.700975000	-0.442188000	1.380236000
1	-4.286564000	-0.950285000	-0.265475000
1	-3.447600000	1.380995000	0.286012000
6	-0.255856000	-3.751689000	3.126101000
1	-1.183893000	-4.051563000	3.630876000
1	0.268522000	-4.653618000	2.763171000
1	0.379634000	-3.228453000	3.848372000
6	-0.964005000	1.492368000	1.679356000
6	0.170304000	0.679616000	1.010439000
1	-0.240588000	-0.113317000	0.380059000
1	-0.634305000	1.883404000	2.648409000
1	-1.186368000	2.358323000	1.033670000
1	0.740585000	1.364623000	0.361593000
1	-1.836439000	-1.913917000	-0.316015000
1	-3.066783000	-3.198734000	-0.361779000
6	-2.388261000	-2.635432000	0.301791000
6	-1.424595000	-3.582193000	0.996984000
1	-1.978044000	-4.368419000	1.524515000
1	-0.786827000	-4.088626000	0.254062000
8	-1.167581000	-0.990233000	4.463022000
1	-0.162207000	-1.167141000	4.762156000
8	1.135115000	-1.580534000	5.173612000
15	2.201562000	-0.644710000	5.855756000
8	3.560448000	-0.693398000	5.167792000
8	1.603240000	0.769179000	6.093307000
8	2.467908000	-1.299108000	7.362346000
1	1.694806000	-1.146925000	7.928312000
1	0.196027000	0.988626000	5.574478000
8	-0.727304000	1.183147000	5.164405000
1	-1.360625000	0.818007000	5.799989000

^{2}**TS1**

E(UM06L) = -1684.69387247

29	-0.133469000	-2.103765000	2.600587000
6	-3.567731000	-4.227191000	3.127027000
1	-3.838662000	-5.156339000	2.608020000
1	-2.748972000	-4.437955000	3.836562000
1	-4.414079000	-3.833515000	3.703182000
6	-3.433146000	-1.852259000	2.469568000
6	-2.638985000	-0.748655000	1.789706000
1	-2.470609000	-0.982394000	0.731543000
6	-1.515379000	0.272823000	3.712443000
1	-2.111148000	-0.345820000	4.390226000
1	-0.529588000	0.429813000	4.170623000
1	-2.013478000	1.249731000	3.574111000
6	2.294311000	-0.594303000	1.845745000
1	2.876423000	-1.326353000	2.422072000
1	2.956736000	-0.109236000	1.104810000
1	1.927106000	0.167670000	2.545018000
6	1.637131000	-2.368356000	0.315131000
1	1.950586000	-1.976845000	-0.669343000
1	2.529712000	-2.805729000	0.781696000
6	0.572932000	-3.441017000	0.122150000
1	1.001054000	-4.281844000	-0.454532000
1	-0.256798000	-3.038585000	-0.473788000
7	0.024824000	-3.921918000	1.411204000
7	-3.102663000	-3.242505000	2.182452000
7	-1.332580000	-0.407067000	2.414014000
7	1.165168000	-1.276029000	1.192659000
1	-3.418299000	-1.751194000	3.566452000
1	-4.497586000	-1.741069000	2.178807000
1	-3.280218000	0.150904000	1.808142000
6	0.978161000	-4.783256000	2.130434000
1	0.570597000	-5.018817000	3.121685000
1	1.173927000	-5.721832000	1.579196000
1	1.928271000	-4.252815000	2.274495000
6	-0.553648000	0.473054000	1.493696000
6	0.267698000	-0.327586000	0.494321000
1	-0.399342000	-0.903568000	-0.160943000
1	0.107759000	1.093433000	2.111055000
1	-1.230421000	1.164033000	0.963056000
1	0.845870000	0.361114000	-0.148502000
1	-2.180185000	-2.856546000	0.344688000
1	-3.221327000	-4.291380000	0.421901000
6	-2.454596000	-3.712067000	0.967625000
6	-1.250542000	-4.636289000	1.209935000
1	-1.435328000	-5.269817000	2.085718000
1	-1.168582000	-5.314976000	0.342444000
8	-0.724147000	-3.014120000	4.145698000
8	-0.237469000	-4.028679000	5.574682000
1	0.057930000	-3.280248000	6.123066000
1	-2.475640000	-2.355427000	8.286849000
8	-3.372107000	-2.636015000	8.039959000
15	-3.448509000	-3.046855000	6.450546000
8	-4.911617000	-3.229223000	6.128807000
8	-2.687747000	-4.481018000	6.362945000
8	-2.617045000	-2.026212000	5.653065000
1	-1.475595000	-2.583396000	4.672556000
1	-1.717080000	-4.353903000	6.104888000

^{2}PC1

E(UM06L) = -1684.74600776

29	-1.332895000	-0.604674000	2.591199000
6	-3.841750000	-2.224656000	2.745809000
1	-3.222570000	-2.931841000	3.311783000
1	-4.244080000	-1.487246000	3.451784000
1	-4.678018000	-2.773801000	2.276762000
6	-3.808970000	-0.476170000	1.036571000
6	-2.904784000	0.676070000	0.629480000
1	-2.171429000	0.332424000	-0.113036000
6	-3.038717000	1.919658000	2.733969000
1	-3.825229000	1.252733000	3.108074000
1	-2.453013000	2.262001000	3.593231000
1	-3.511361000	2.787166000	2.238593000
6	0.997670000	1.067329000	3.432690000
1	0.224044000	1.763268000	3.774894000
1	1.221986000	0.377312000	4.251752000
1	1.911385000	1.635660000	3.181491000
6	1.469936000	-0.778983000	1.901052000
1	2.241655000	-0.400124000	1.209857000
1	1.984135000	-1.098388000	2.816130000
6	0.734879000	-1.953912000	1.274197000
1	1.444516000	-2.771472000	1.055414000
1	0.278662000	-1.649695000	0.322036000
7	-0.350839000	-2.436307000	2.165537000
7	-3.030653000	-1.529644000	1.733783000
7	-2.157922000	1.198946000	1.799537000
7	0.519945000	0.302743000	2.267660000
1	-4.592552000	-0.122723000	1.718205000
1	-4.318147000	-0.889677000	0.149614000
1	-3.499749000	1.480694000	0.161577000
6	0.180574000	-3.160171000	3.333072000
1	-0.645377000	-3.414014000	4.008483000
1	0.687790000	-4.088351000	3.012698000
1	0.887666000	-2.539881000	3.892018000
6	-1.007256000	2.040482000	1.385193000
6	0.218103000	1.183121000	1.110704000
1	0.039832000	0.543226000	0.235607000
1	-0.796549000	2.751051000	2.194285000
1	-1.259242000	2.636661000	0.491806000
1	1.087797000	1.824522000	0.882409000
1	-1.960387000	-1.854041000	-0.039913000
1	-3.160446000	-3.117451000	0.311602000
6	-2.398984000	-2.458428000	0.766013000
6	-1.320453000	-3.292478000	1.433949000
1	-1.770710000	-3.984367000	2.156623000
1	-0.800673000	-3.910683000	0.682672000
8	-1.942688000	-0.500351000	4.750257000
1	-1.375220000	-1.137265000	5.339464000
8	-0.503833000	-1.907727000	6.272464000
15	0.856538000	-1.285216000	6.630704000
8	2.053601000	-1.552507000	5.747597000
8	0.628622000	0.324493000	6.812865000
8	1.282098000	-1.750705000	8.137083000
1	0.540858000	-1.660494000	8.759300000
1	-0.210340000	0.594604000	6.370582000
8	-1.665418000	0.773691000	5.362966000
1	-2.485513000	0.952742000	5.858827000

^{2}E

E(UM06L) = -1040.86537185

29	0.016222000	−1.341752000	2.309886000
6	−2.567778000	−2.773083000	2.599511000
1	−1.949042000	−3.611143000	2.944079000
1	−2.781750000	−2.130432000	3.463099000
1	−3.516876000	−3.170187000	2.197234000
6	−2.599529000	−0.778282000	1.171663000
6	−1.651037000	0.387324000	0.922171000
1	−1.027436000	0.179070000	0.041044000
6	−1.462401000	1.067538000	3.277553000
1	−2.249528000	0.358021000	3.559897000
1	−0.759946000	1.136418000	4.115765000
1	−1.918843000	2.056438000	3.093135000
6	2.590718000	−0.124473000	3.201063000
1	2.735422000	−1.017794000	3.821838000
1	3.572661000	0.331289000	2.981429000
1	1.982742000	0.587743000	3.768926000
6	2.683851000	−1.517192000	1.196892000
1	3.324897000	−1.024897000	0.447280000
1	3.353813000	−2.028337000	1.898808000
6	1.766176000	−2.523271000	0.516886000
1	2.362595000	−3.331030000	0.057011000
1	1.197574000	−2.028933000	−0.282988000
7	0.785872000	−3.086485000	1.480705000
7	−1.845005000	−1.990276000	1.583942000
7	−0.745765000	0.587305000	2.082902000
7	1.900294000	−0.508628000	1.958662000
1	−3.310079000	−0.528947000	1.969285000
1	−3.195605000	−0.980021000	0.266613000
1	−2.225388000	1.308340000	0.717661000
6	1.415677000	−3.947612000	2.495455000
1	0.645673000	−4.300893000	3.194475000
1	1.909453000	−4.819342000	2.030373000
1	2.161078000	−3.381119000	3.067818000
6	0.407785000	1.462156000	1.752092000
6	1.540178000	0.663719000	1.120191000
1	1.230759000	0.285468000	0.136078000
1	0.750697000	1.931672000	2.682763000
1	0.097536000	2.277050000	1.076896000
1	2.420278000	1.312739000	0.965980000
1	−1.075829000	−2.115544000	−0.361854000
1	−2.299717000	−3.352889000	0.002188000
6	−1.433474000	−2.805818000	0.414868000
6	−0.330888000	−3.784343000	0.791934000
1	−0.721294000	−4.549745000	1.474148000
1	0.033237000	−4.309994000	−0.106364000
8	−0.034531000	−1.744329000	4.570842000
8	0.357392000	−0.666340000	5.436352000
1	−0.419300000	−0.600041000	6.021420000
1	0.633236000	−2.425859000	4.771690000

^{4}RC2

E(UM06L) = -1684.24881538

29	−0.843865000	−1.331818000	2.515510000
6	−4.112841000	−2.730862000	2.025033000
1	−4.274572000	−3.818090000	2.026455000
1	−3.378390000	−2.493625000	2.861963000
1	−5.039987000	−2.179101000	2.225321000
6	−3.758763000	−0.962324000	0.306435000
6	−2.539375000	−0.042661000	0.264689000
1	−1.768058000	−0.494104000	−0.368560000
6	−2.919195000	0.931480000	2.478230000
1	−3.659142000	0.179508000	2.779025000
1	−2.399427000	1.271125000	3.380117000
1	−3.438495000	1.786095000	2.006857000
6	1.687743000	−0.030563000	2.695042000
1	1.915861000	−0.833996000	3.404679000
1	2.622899000	0.375882000	2.271812000
1	1.160796000	0.765246000	3.234460000
6	1.488631000	−1.679995000	0.898149000
1	2.106559000	−1.297899000	0.069184000
1	2.166887000	−2.170195000	1.606968000
6	0.464634000	−2.673387000	0.370256000
1	0.981178000	−3.526878000	−0.104788000
1	−0.153180000	−2.192981000	−0.398874000
7	−0.430912000	−3.154788000	1.452795000
7	−3.513148000	−2.299363000	0.815923000
7	−1.933827000	0.334857000	1.560347000
7	0.824082000	−0.558423000	1.614491000
1	−4.585272000	−0.522335000	0.874234000
1	−4.092551000	−1.073851000	−0.740349000
1	−2.883187000	0.873145000	−0.252086000
6	0.286982000	−4.020306000	2.413913000
1	−0.407699000	−4.318078000	3.209251000
1	0.674585000	−4.923650000	1.908609000
1	1.105891000	−3.479624000	2.900218000
6	−0.807134000	1.267608000	1.303297000
6	0.385709000	0.534622000	0.708937000
1	0.135276000	0.104225000	−0.268576000
1	−0.530279000	1.721239000	2.263580000
1	−1.123214000	2.084524000	0.629920000
1	1.216350000	1.240403000	0.543044000
1	−2.168234000	−2.634750000	−0.745216000
1	−3.261074000	−3.966585000	−0.364842000
6	−2.619163000	−3.187525000	0.085708000
6	−1.579494000	−3.928138000	0.926279000
1	−2.069485000	−4.408468000	1.781982000
1	−1.190640000	−4.741715000	0.287646000
8	−1.989560000	−1.967891000	3.826927000
8	−0.940995000	0.450231000	5.067422000
1	−1.529564000	−0.286412000	4.833852000
1	−0.127291000	−0.037804000	5.423584000
8	1.131092000	−0.771646000	6.011572000
15	1.322933000	−2.317302000	5.917471000
8	1.776090000	−2.972641000	7.226105000
8	2.157074000	−2.721618000	4.676027000
8	−0.226749000	−2.945069000	5.691369000
1	−0.757425000	−2.420564000	5.052398000

^{4}INT2a

E(UM06L) = -1684.24694932

29	-0.400830000	-1.335797000	3.095186000
6	-4.008010000	-2.535565000	2.534030000
1	-3.563149000	-3.499418000	2.794898000
1	-4.017118000	-1.863957000	3.403761000
1	-5.057688000	-2.685430000	2.211149000
6	-3.753839000	-0.576622000	1.043425000
6	-2.624164000	0.438157000	0.901413000
1	-1.949421000	0.118281000	0.097648000
6	-2.676577000	1.189227000	3.205049000
1	-3.412952000	0.447555000	3.546905000
1	-2.038953000	1.439926000	4.060642000
1	-3.220805000	2.105393000	2.896036000
6	2.047707000	0.280293000	2.889800000
1	2.376688000	-0.493040000	3.595292000
1	2.912657000	0.659489000	2.316623000
1	1.606405000	1.106395000	3.457634000
6	1.666495000	-1.343366000	1.130217000
1	2.239191000	-0.885316000	0.305118000
1	2.381360000	-1.890505000	1.757636000
6	0.621477000	-2.293451000	0.569489000
1	1.109568000	-3.081210000	-0.031191000
1	-0.055311000	-1.748317000	-0.099852000
7	-0.181206000	-2.901728000	1.658825000
7	-3.310182000	-1.900874000	1.449471000
7	-1.850404000	0.646663000	2.125241000
7	1.038757000	-0.295376000	1.974742000
1	-4.522656000	-0.244540000	1.749651000
1	-4.233482000	-0.693781000	0.053595000
1	-3.097497000	1.381555000	0.556770000
6	0.601141000	-3.939354000	2.370743000
1	-0.000277000	-4.336422000	3.196156000
1	0.868881000	-4.759574000	1.680893000
1	1.503004000	-3.506637000	2.814558000
6	-0.692805000	1.510412000	1.852337000
6	0.428800000	0.770528000	1.139729000
1	0.048170000	0.318065000	0.215672000
1	-0.325843000	1.898845000	2.811283000
1	-0.981460000	2.390701000	1.242516000
1	1.211820000	1.487851000	0.838572000
1	-2.070566000	-1.989214000	-0.219356000
1	-3.182052000	-3.343465000	0.012341000
6	-2.477520000	-2.667675000	0.537875000
6	-1.406740000	-3.558771000	1.152447000
1	-1.829948000	-4.146069000	1.975972000
1	-1.121143000	-4.279527000	0.365419000
8	-1.509678000	-2.395694000	4.204394000
8	-0.234364000	-0.091840000	4.731089000
1	-1.097721000	-0.144534000	5.169924000
1	0.440649000	-0.603141000	5.451298000
8	1.201421000	-1.206989000	6.356077000
15	1.750367000	-2.628499000	5.940859000
8	2.507859000	-3.313099000	7.076298000
8	2.495795000	-2.551147000	4.586524000
8	0.389576000	-3.562533000	5.711923000
1	-0.282816000	-3.116070000	5.125971000

^{4}INT2b

E(UM06L) = -1684.25169593

29	-0.469293000	-1.261932000	3.127307000
6	-3.850139000	-2.418406000	2.477716000
1	-4.738874000	-1.871268000	2.811975000
1	-4.056814000	-3.496476000	2.378805000
1	-3.039421000	-2.333814000	3.265415000
6	-3.831970000	-0.602445000	0.757996000
6	-2.733253000	0.457731000	0.672101000
1	-2.013413000	0.164554000	-0.103333000
6	-2.891446000	1.187505000	2.980198000
1	-3.654486000	0.445287000	3.255207000
1	-2.292557000	1.387038000	3.876369000
1	-3.409487000	2.123361000	2.681806000
6	1.896528000	0.480580000	2.891797000
1	2.250395000	-0.246374000	3.632711000
1	2.753265000	0.884920000	2.322974000
1	1.399663000	1.300968000	3.421102000
6	1.628767000	-1.240553000	1.205944000
1	2.229734000	-0.799401000	0.391097000
1	2.324426000	-1.747118000	1.886852000
6	0.633980000	-2.235783000	0.629997000
1	1.170612000	-3.030619000	0.082355000
1	-0.020100000	-1.732960000	-0.092494000
7	-0.211552000	-2.828534000	1.696021000
7	-3.361527000	-1.885159000	1.256115000
7	-2.017846000	0.675460000	1.926069000
7	0.939321000	-0.179573000	1.981107000
1	-4.664097000	-0.276721000	1.391582000
1	-4.225007000	-0.765515000	-0.261114000
1	-3.223259000	1.387704000	0.311224000
6	0.549524000	-3.852158000	2.452848000
1	1.434171000	-3.407746000	2.919424000
1	-0.079770000	-4.242833000	3.261113000
1	0.847192000	-4.681961000	1.787124000
6	-0.860016000	1.551156000	1.712159000
6	0.315857000	0.821900000	1.078612000
1	-0.013088000	0.315216000	0.162740000
1	-0.554943000	1.958917000	2.684393000
1	-1.123853000	2.419377000	1.073440000
1	1.082603000	1.554851000	0.771812000
1	-2.002454000	-1.982439000	-0.315926000
1	-3.089721000	-3.356969000	-0.147578000
6	-2.450769000	-2.656085000	0.423540000
6	-1.406859000	-3.508276000	1.137498000
1	-1.871955000	-4.076164000	1.952462000
1	-1.063699000	-4.250551000	0.394653000
8	-1.536527000	-2.393478000	4.254064000
8	-0.420411000	0.000260000	4.651128000
1	-1.269161000	-0.116710000	5.105112000
1	0.505832000	-0.577539000	5.659889000
8	1.108280000	-0.981968000	6.407376000
15	1.705540000	-2.432485000	5.992558000
8	2.501743000	-2.987059000	7.156765000
8	2.396857000	-2.320777000	4.627090000
8	0.365326000	-3.376687000	5.844264000
1	-0.323604000	-2.987834000	5.231245000

MECPb

E(UM06L) = -1684.24499179

29	-0.332052000	-1.714956000	2.915143000
6	-3.976346000	-2.755428000	2.380903000
1	-3.474358000	-3.699558000	2.603922000
1	-4.026054000	-2.124841000	3.279966000
1	-5.016155000	-2.951741000	2.051586000
6	-3.851407000	-0.711203000	0.996160000
6	-2.783242000	0.359057000	0.815983000
1	-2.153159000	0.103369000	-0.045810000
6	-2.657486000	1.101078000	3.125963000
1	-3.503589000	0.464512000	3.423055000
1	-1.981733000	1.169706000	3.987291000
1	-3.053218000	2.113910000	2.900454000
6	1.973555000	0.042618000	2.708342000
1	2.361959000	-0.722412000	3.391073000
1	2.805374000	0.496478000	2.141365000
1	1.483831000	0.817295000	3.307056000
6	1.674354000	-1.575904000	0.926655000
1	2.261158000	-1.076392000	0.136066000
1	2.379578000	-2.124560000	1.563798000
6	0.670441000	-2.534704000	0.307210000
1	1.197119000	-3.310008000	-0.275431000
1	0.015734000	-2.001593000	-0.393221000
7	-0.163728000	-3.156187000	1.363843000
7	-3.315445000	-2.028274000	1.329316000
7	-1.939035000	0.523841000	1.994989000
7	0.995615000	-0.572843000	1.787741000
1	-4.573595000	-0.440513000	1.774439000
1	-4.408699000	-0.826712000	0.047313000
1	-3.308881000	1.299352000	0.542195000
6	0.557601000	-4.276123000	2.008129000
1	-0.018591000	-4.615150000	2.876970000
1	0.695506000	-5.110359000	1.297458000
1	1.529959000	-3.938035000	2.378680000
6	-0.727146000	1.283884000	1.684483000
6	0.333356000	0.467400000	0.957163000
1	-0.117378000	-0.022720000	0.086044000
1	-0.317103000	1.676180000	2.624414000
1	-0.961473000	2.169975000	1.058229000
1	1.109337000	1.150548000	0.568697000
1	-2.044802000	-1.943011000	-0.310981000
1	-3.197204000	-3.275985000	-0.274776000
6	-2.480872000	-2.701787000	0.347159000
6	-1.437341000	-3.698290000	0.844374000
1	-1.864136000	-4.341626000	1.623207000
1	-1.214223000	-4.355873000	-0.013904000
8	-1.352488000	-2.790619000	4.054608000
8	-0.286255000	-0.597143000	4.467971000
1	-1.111985000	-0.753109000	4.952402000
1	0.763671000	-1.219639000	5.438590000
8	1.408127000	-1.648334000	6.100886000
15	1.883818000	-3.125484000	5.589365000
8	2.682419000	-3.770844000	6.705098000
8	2.550939000	-2.976406000	4.214423000
8	0.483683000	-3.952127000	5.437669000
1	-0.232643000	-3.470910000	4.876066000

^{2}INT2b

E(UM06L) = -1684.25695304

29	-0.268512000	-1.710120000	2.854954000
6	-4.283355000	-2.928105000	2.078545000
1	-3.844550000	-3.892362000	2.372701000
1	-4.720377000	-2.475493000	2.982043000
1	-5.110018000	-3.138148000	1.363771000
6	-3.823315000	-0.748354000	1.152558000
6	-2.781874000	0.346573000	0.928061000
1	-2.169494000	0.089593000	0.052578000
6	-2.585025000	1.209438000	3.181740000
1	-3.401079000	0.567437000	3.545003000
1	-1.886629000	1.361631000	4.016310000
1	-3.021471000	2.196893000	2.914306000
6	2.013956000	0.027891000	2.693583000
1	2.395572000	-0.735944000	3.380684000
1	2.837945000	0.439690000	2.088017000
1	1.548449000	0.831493000	3.270234000
6	1.686958000	-1.601764000	0.916511000
1	2.250189000	-1.089660000	0.120200000
1	2.404770000	-2.147925000	1.539927000
6	0.651196000	-2.532595000	0.334805000
1	1.126484000	-3.340488000	-0.242829000
1	-0.023273000	-1.997009000	-0.340961000
7	-0.161627000	-3.128534000	1.439183000
7	-3.272571000	-2.049035000	1.516271000
7	-1.895062000	0.584056000	2.063518000
7	1.017172000	-0.589875000	1.789979000
1	-4.512689000	-0.444702000	1.954521000
1	-4.443628000	-0.814292000	0.230345000
1	-3.327695000	1.274733000	0.646520000
6	0.589554000	-4.241290000	2.083255000
1	-0.019944000	-4.665753000	2.886658000
1	0.797718000	-5.016566000	1.329133000
1	1.521293000	-3.871718000	2.522670000
6	-0.688858000	1.310877000	1.690445000
6	0.329945000	0.446118000	0.960500000
1	-0.152737000	-0.059901000	0.117914000
1	-0.237836000	1.733568000	2.598115000
1	-0.911826000	2.174348000	1.027901000
1	1.120062000	1.090439000	0.540249000
1	-2.022336000	-1.909151000	-0.157622000
1	-3.173047000	-3.235585000	-0.240638000
6	-2.506310000	-2.680661000	0.453785000
6	-1.461728000	-3.676861000	0.945923000
1	-1.871739000	-4.285070000	1.761514000
1	-1.204907000	-4.363344000	0.121585000
8	-1.302868000	-2.757601000	3.918572000
8	-0.200110000	-0.560238000	4.302978000
1	-1.092528000	-0.561311000	4.689214000
1	0.794534000	-1.219149000	5.410985000
8	1.379240000	-1.643236000	6.108065000
15	1.956987000	-3.079962000	5.586451000
8	2.780903000	-3.689317000	6.696883000
8	2.586947000	-2.887669000	4.204966000
8	0.596568000	-3.998085000	5.441530000
1	-0.072111000	-3.590107000	4.835681000

^{4}INT2c

E(UM06L) = -1684.24837832

29	-0.965993000	-1.219402000	2.810154000
6	-3.812372000	-2.621651000	2.603921000
1	-3.297486000	-3.531568000	2.931880000
1	-4.063476000	-2.031243000	3.494904000
1	-4.745948000	-2.905794000	2.082938000
6	-3.632146000	-0.597177000	1.280799000
6	-2.625854000	0.481865000	0.950415000
1	-1.993803000	0.167740000	0.110048000
6	-2.505664000	1.384444000	3.213485000
1	-3.252134000	0.685882000	3.611323000
1	-1.816281000	1.638755000	4.022953000
1	-3.017299000	2.297624000	2.858729000
6	1.781142000	0.191691000	3.084086000
1	2.055549000	-0.620659000	3.764516000
1	2.696914000	0.633771000	2.650176000
1	1.245510000	0.960364000	3.648717000
6	1.631819000	-1.415780000	1.269929000
1	2.315914000	-0.974203000	0.523905000
1	2.250207000	-1.967212000	1.987602000
6	0.646432000	-2.340028000	0.589531000
1	1.187594000	-3.121937000	0.026367000
1	0.033303000	-1.785382000	-0.131666000
7	-0.254068000	-2.971843000	1.579540000
7	-2.950588000	-1.831427000	1.720037000
7	-1.753385000	0.763510000	2.111846000
7	0.931431000	-0.339088000	2.007650000
1	-4.303806000	-0.268474000	2.083272000
1	-4.268131000	-0.801545000	0.400668000
1	-3.146268000	1.406195000	0.639618000
6	0.465389000	-3.938857000	2.425441000
1	-0.242218000	-4.428049000	3.102243000
1	0.942971000	-4.705933000	1.788278000
1	1.226345000	-3.442001000	3.035571000
6	-0.589505000	1.588806000	1.727509000
6	0.479665000	0.732801000	1.087088000
1	0.092776000	0.267436000	0.172259000
1	-0.202596000	2.078560000	2.629418000
1	-0.900608000	2.389152000	1.033190000
1	1.344380000	1.354054000	0.793977000
1	-2.074100000	-1.906786000	-0.186732000
1	-3.328565000	-3.127218000	0.078084000
6	-2.477416000	-2.607386000	0.555439000
6	-1.418774000	-3.616279000	0.940719000
1	-1.827181000	-4.347728000	1.648744000
1	-1.094568000	-4.177865000	0.046429000
8	-1.314408000	-2.543042000	4.163721000
8	-0.639027000	-0.334144000	4.612694000
1	-1.484185000	-0.394051000	5.085707000
1	0.486864000	-0.858624000	5.540940000
8	1.202859000	-1.130112000	6.215748000
15	1.826911000	-2.612958000	5.942659000
8	2.535232000	-3.063620000	7.204275000
8	2.624244000	-2.606362000	4.635003000
8	0.493369000	-3.549069000	5.757931000
1	-0.142218000	-3.170559000	5.074802000

^{2}INT2c

E(UM06L) = -1684.23919027

29	-0.979303000	-1.240867000	2.845657000
6	-3.804950000	-2.633131000	2.601618000
1	-3.275592000	-3.532540000	2.935918000
1	-4.067050000	-2.038721000	3.486129000
1	-4.732068000	-2.934532000	2.078646000
6	-3.635561000	-0.600377000	1.280229000
6	-2.624860000	0.474064000	0.953500000
1	-1.990433000	0.158838000	0.115692000
6	-2.512343000	1.365310000	3.223035000
1	-3.253827000	0.661546000	3.620915000
1	-1.823983000	1.625659000	4.031082000
1	-3.030109000	2.274489000	2.867175000
6	1.805651000	0.185643000	3.073236000
1	2.081664000	-0.628098000	3.751650000
1	2.721076000	0.627572000	2.637313000
1	1.274163000	0.955010000	3.641025000
6	1.641663000	-1.416889000	1.255237000
1	2.323705000	-0.976189000	0.506128000
1	2.262699000	-1.972703000	1.967219000
6	0.649140000	-2.334157000	0.576891000
1	1.184324000	-3.120736000	0.014280000
1	0.039873000	-1.776801000	-0.145662000
7	-0.256484000	-2.957093000	1.566895000
7	-2.956159000	-1.838127000	1.711990000
7	-1.756981000	0.751483000	2.119968000
7	0.953382000	-0.339977000	1.999353000
1	-4.307547000	-0.274425000	2.083199000
1	-4.270245000	-0.802336000	0.398658000
1	-3.138633000	1.401866000	0.642534000
6	0.449370000	-3.912412000	2.433908000
1	-0.264915000	-4.369717000	3.125654000
1	0.912900000	-4.703104000	1.815204000
1	1.218861000	-3.413959000	3.031604000
6	-0.589255000	1.576209000	1.745472000
6	0.481009000	0.731387000	1.091757000
1	0.092224000	0.269565000	0.176116000
1	-0.202297000	2.055319000	2.652882000
1	-0.902256000	2.384799000	1.061475000
1	1.336558000	1.364513000	0.795844000
1	-2.075353000	-1.900244000	-0.194278000
1	-3.327693000	-3.124221000	0.064314000
6	-2.476660000	-2.606248000	0.543568000
6	-1.416179000	-3.610318000	0.928048000
1	-1.821204000	-4.343915000	1.635754000
1	-1.088350000	-4.171476000	0.034806000
8	-1.350046000	-2.537679000	4.220620000
8	-0.621470000	-0.337589000	4.578657000
1	-1.447525000	-0.390333000	5.084277000
1	0.507094000	-0.843919000	5.521087000
8	1.210925000	-1.116043000	6.205162000
15	1.810787000	-2.613305000	5.941754000
8	2.514949000	-3.062079000	7.207477000
8	2.616195000	-2.619789000	4.637857000
8	0.467122000	-3.526080000	5.759196000
1	-0.199256000	-3.125547000	5.100753000

^{2}TS2

E(UM06L) = -1684.23413512

29	-0.972695000	-1.232762000	2.698691000
6	-3.700480000	-2.721189000	2.598721000
1	-3.111497000	-3.591531000	2.909653000
1	-3.997789000	-2.174323000	3.501678000
1	-4.606292000	-3.066636000	2.067105000
6	-3.660355000	-0.613918000	1.396141000
6	-2.726926000	0.513238000	1.006255000
1	-2.164809000	0.249588000	0.100703000
6	-2.399792000	1.465109000	3.229212000
1	-3.188812000	0.830124000	3.652273000
1	-1.653764000	1.648455000	4.010302000
1	-2.844634000	2.428455000	2.918536000
6	1.673250000	0.271268000	3.091793000
1	1.956218000	-0.495810000	3.817668000
1	2.584152000	0.743059000	2.680292000
1	1.078525000	1.030656000	3.608945000
6	1.668356000	-1.426285000	1.356936000
1	2.402556000	-0.995375000	0.653443000
1	2.232191000	-1.947335000	2.139709000
6	0.753266000	-2.389085000	0.628940000
1	1.348088000	-3.194506000	0.162146000
1	0.215093000	-1.876362000	-0.178897000
7	-0.245456000	-2.969017000	1.555024000
7	-2.905900000	-1.840249000	1.736451000
7	-1.759316000	0.799610000	2.085940000
7	0.891061000	-0.345379000	2.008519000
1	-4.258190000	-0.330326000	2.271539000
1	-4.370102000	-0.818574000	0.574679000
1	-3.312632000	1.420183000	0.768229000
6	0.362081000	-3.960293000	2.456041000
1	-0.400095000	-4.335411000	3.147170000
1	0.766656000	-4.804213000	1.867702000
1	1.162654000	-3.508529000	3.049739000
6	-0.606859000	1.581306000	1.589655000
6	0.449311000	0.662552000	1.011734000
1	0.044008000	0.128284000	0.142815000
1	-0.195606000	2.159658000	2.425940000
1	-0.930303000	2.310160000	0.826064000
1	1.318354000	1.249059000	0.663542000
1	-2.021550000	-1.768656000	-0.166027000
1	-3.291882000	-2.996754000	-0.008651000
6	-2.437481000	-2.525571000	0.510864000
6	-1.387229000	-3.567116000	0.827680000
1	-1.811780000	-4.361129000	1.453943000
1	-1.038702000	-4.047145000	-0.102908000
8	-1.357136000	-2.384213000	4.273496000
8	-0.693112000	-0.497932000	4.635802000
1	-1.556996000	-0.362058000	5.055719000
1	0.532553000	-0.911166000	5.618541000
8	1.276782000	-1.171200000	6.238662000
15	1.834112000	-2.681811000	5.914292000
8	2.528077000	-3.195310000	7.159278000
8	2.632294000	-2.661869000	4.608387000
8	0.455073000	-3.533737000	5.707101000
1	-0.199792000	-3.070395000	5.081269000

^{4}TS2

E(UM06L) = -1684.23553925

29	-1.003764000	-1.171554000	2.449579000
6	-3.698514000	-2.619752000	2.628842000
1	-3.130434000	-3.495316000	2.962866000
1	-3.915493000	-2.000828000	3.508732000
1	-4.648353000	-2.955032000	2.174479000
6	-3.653340000	-0.620583000	1.240661000
6	-2.690868000	0.505025000	0.910306000
1	-2.079118000	0.232981000	0.039032000
6	-2.473143000	1.350504000	3.203850000
1	-3.233336000	0.653977000	3.578895000
1	-1.749328000	1.521937000	4.007215000
1	-2.958654000	2.303707000	2.927779000
6	1.701547000	0.217277000	3.033308000
1	1.930585000	-0.587990000	3.740177000
1	2.642767000	0.640981000	2.638196000
1	1.153029000	0.998640000	3.567078000
6	1.630995000	-1.391770000	1.219232000
1	2.310794000	-0.957772000	0.466182000
1	2.255017000	-1.908395000	1.958436000
6	0.680521000	-2.370695000	0.555615000
1	1.250342000	-3.175234000	0.058446000
1	0.085354000	-1.862066000	-0.214704000
7	-0.257146000	-2.959753000	1.545766000
7	-2.914303000	-1.833784000	1.666332000
7	-1.772968000	0.767848000	2.046614000
7	0.888897000	-0.318745000	1.926421000
1	-4.323626000	-0.324427000	2.057077000

1	-4.291585000	-0.841280000	0.368057000
1	-3.249552000	1.422010000	0.650865000
6	0.425077000	-3.897225000	2.456616000
1	-0.305679000	-4.312581000	3.157866000
1	0.879755000	-4.718748000	1.874509000
1	1.197509000	-3.386368000	3.039544000
6	-0.626828000	1.610982000	1.625203000
6	0.460080000	0.757376000	0.995337000
1	0.081414000	0.287291000	0.077882000
1	-0.240387000	2.128200000	2.512010000
1	-0.958458000	2.388671000	0.916562000
1	1.325127000	1.382848000	0.713267000
1	-2.106841000	-1.944858000	-0.268423000
1	-3.346702000	-3.171619000	0.060906000
6	-2.484771000	-2.635813000	0.497304000
6	-1.404981000	-3.628537000	0.883106000
1	-1.804730000	-4.373350000	1.581779000
1	-1.057777000	-4.175838000	-0.009699000
8	-1.198891000	-2.121372000	4.215592000
8	-0.480383000	-0.290190000	5.050151000
1	-1.412846000	-0.267765000	5.318138000
1	0.563489000	-0.937007000	6.030004000
8	1.269291000	-1.350516000	6.632756000
15	1.800683000	-2.778000000	6.044417000
8	2.502478000	-3.519343000	7.163666000
8	2.565823000	-2.542938000	4.738563000
8	0.403210000	-3.574614000	5.716500000
1	-0.174239000	-3.031004000	5.099595000

^{4}INT2d

E(UM06L) = -1684.23321253

29	-1.023395000	-1.234501000	2.305449000
6	-3.597482000	-2.715349000	2.541195000
1	-2.957971000	-3.553286000	2.843382000
1	-3.828007000	-2.125073000	3.437531000
1	-4.537150000	-3.113582000	2.117481000
6	-3.687778000	-0.643998000	1.250076000
6	-2.770837000	0.523400000	0.922450000
1	-2.229560000	0.324857000	-0.012894000
6	-2.364900000	1.281503000	3.221720000
1	-3.184913000	0.639106000	3.566582000
1	-1.607918000	1.305740000	4.014045000
1	-2.756957000	2.299432000	3.045312000
6	1.566283000	0.135397000	3.021574000
1	1.816696000	-0.684116000	3.703579000
1	2.494252000	0.646058000	2.706118000
1	0.930362000	0.845321000	3.560705000
6	1.639944000	-1.473980000	1.190201000
1	2.361087000	-1.040595000	0.475943000
1	2.220294000	-1.983698000	1.968850000
6	0.735105000	-2.466744000	0.476689000
1	1.339474000	-3.290497000	0.056389000
1	0.220203000	-1.976160000	-0.360885000
7	-0.302538000	-3.001869000	1.390776000
7	-2.898980000	-1.859113000	1.568485000
7	-1.767825000	0.727204000	1.995463000
7	0.851682000	-0.403625000	1.849650000
1	-4.310120000	-0.406680000	2.121965000
1	-4.374634000	-0.835715000	0.408300000
1	-3.365761000	1.442000000	0.771711000
6	0.246020000	-3.940536000	2.384531000
1	-0.551791000	-4.234188000	3.078166000
1	0.647740000	-4.842669000	1.888087000
1	1.036985000	-3.465853000	2.973937000
6	-0.625843000	1.550122000	1.520920000
6	0.452049000	0.669647000	0.904827000
1	0.071169000	0.192448000	-0.008854000
1	-0.222420000	2.102253000	2.378424000
1	-0.965522000	2.301411000	0.788687000
1	1.329470000	1.278960000	0.622972000
1	-2.123985000	-1.850181000	-0.379578000
1	-3.383127000	-3.079487000	-0.118055000
6	-2.504692000	-2.589109000	0.338975000
6	-1.429190000	-3.617693000	0.643660000
1	-1.841611000	-4.428349000	1.256783000
1	-1.069736000	-4.079537000	-0.290936000
8	-1.044797000	-1.679082000	4.340016000
8	-0.388585000	-0.147440000	5.657707000
1	-1.007090000	-0.583632000	6.269170000
1	0.896552000	-0.949486000	6.206319000
8	1.628819000	-1.485691000	6.653742000
15	1.817220000	-2.958426000	5.972260000
8	2.476411000	-3.870275000	6.985115000
8	2.474655000	-2.801632000	4.599869000
8	0.266097000	-3.480710000	5.787792000
1	-0.290488000	-2.833872000	5.273203000

MECPd

E(UM06L) = -1684.23264166

29	-1.027328000	-1.238064000	2.317909000
6	-3.578575000	-2.767179000	2.504370000
1	-2.925506000	-3.597481000	2.798675000
1	-3.833038000	-2.199551000	3.408895000
1	-4.504613000	-3.175979000	2.061422000
6	-3.693432000	-0.671387000	1.255462000
6	-2.793804000	0.511650000	0.938444000
1	-2.248803000	0.328374000	0.002124000
6	-2.400827000	1.263257000	3.241587000
1	-3.215309000	0.610953000	3.581496000
1	-1.645644000	1.291436000	4.035604000
1	-2.802057000	2.278376000	3.067913000
6	1.538018000	0.176273000	3.060884000
1	1.790301000	-0.637262000	3.749097000
1	2.464137000	0.692789000	2.749308000
1	0.892160000	0.885003000	3.589991000
6	1.649038000	-1.433248000	1.233369000
1	2.379347000	-0.987388000	0.536060000
1	2.217823000	-1.943202000	2.020784000
6	0.767962000	-2.429212000	0.494385000
1	1.389804000	-3.240264000	0.074752000
1	0.260702000	-1.937678000	-0.347370000
7	-0.277479000	-2.988737000	1.384215000
7	-2.885978000	-1.878043000	1.556585000
7	-1.795131000	0.720853000	2.013912000
7	0.838223000	-0.374701000	1.885530000
1	-4.314960000	-0.453166000	2.132764000
1	-4.380710000	-0.863791000	0.413924000
1	-3.401736000	1.423002000	0.794550000
6	0.265630000	-3.941625000	2.366774000
1	-0.537657000	-4.253156000	3.046306000
1	0.676885000	-4.832668000	1.858006000
1	1.049149000	-3.473580000	2.971355000
6	-0.662155000	1.559383000	1.545783000
6	0.431485000	0.693098000	0.937236000
1	0.062114000	0.211450000	0.021302000
1	-0.272119000	2.118175000	2.404842000
1	-1.008222000	2.304950000	0.810563000
1	1.303032000	1.313281000	0.660575000
1	-2.089158000	-1.818846000	-0.383038000
1	-3.339238000	-3.065461000	-0.161457000
6	-2.470567000	-2.576617000	0.315513000
6	-1.387667000	-3.600456000	0.609353000
1	-1.797861000	-4.430632000	1.197651000
1	-1.011595000	-4.035387000	-0.331729000
8	-1.076291000	-1.707132000	4.350387000
8	-0.440636000	-0.196878000	5.643632000
1	-1.029767000	-0.636128000	6.281920000
1	0.881509000	-0.974997000	6.171671000
8	1.625416000	-1.495830000	6.614005000
15	1.839431000	-2.962938000	5.925735000
8	2.515112000	-3.869233000	6.932618000
8	2.494474000	-2.784522000	4.555146000
8	0.297033000	-3.508834000	5.740422000
1	-0.277959000	-2.862615000	5.244367000

^{2}TS0

E(UM06L) = -1040.79398350

29	-0.117649000	-2.027087000	1.936045000
6	-2.717789000	-3.472259000	2.133044000
1	-2.109623000	-4.334125000	2.433898000
1	-2.945499000	-2.886312000	3.032380000
1	-3.660912000	-3.836071000	1.687391000
6	-2.733198000	-1.395129000	0.847288000
6	-1.793745000	-0.219567000	0.615114000
1	-1.207196000	-0.381747000	-0.299909000
6	-1.498811000	0.397718000	2.978332000
1	-2.295982000	-0.297553000	3.268372000
1	-0.761544000	0.425746000	3.789582000
1	-1.932006000	1.404958000	2.842417000
6	2.491219000	-0.797739000	2.815019000
1	2.665058000	-1.684585000	3.437923000
1	3.463187000	-0.330950000	2.573742000
1	1.887357000	-0.088016000	3.391085000
6	2.564508000	-2.209764000	0.830108000
1	3.237932000	-1.719800000	0.107164000
1	3.201276000	-2.747144000	1.543511000
6	1.647899000	-3.185366000	0.107943000
1	2.244277000	-3.991667000	-0.355156000
1	1.106888000	-2.668590000	-0.696395000
7	0.637614000	-3.754822000	1.033469000
7	-1.976236000	-2.629045000	1.179881000
7	-0.842607000	-0.066237000	1.744605000
7	1.783623000	-1.196871000	1.587806000
1	-3.410694000	-1.179922000	1.682929000
1	-3.364832000	-1.553902000	-0.042386000
1	-2.378634000	0.707180000	0.476120000
6	1.225757000	-4.660354000	2.034027000
1	0.434451000	-5.012497000	2.708029000
1	1.708551000	-5.528951000	1.551489000
1	1.970686000	-4.127338000	2.637803000
6	0.315732000	0.790448000	1.384723000
6	1.423389000	-0.031475000	0.740051000
1	1.087273000	-0.419206000	-0.231468000
1	0.683181000	1.267042000	2.302200000
1	0.004049000	1.602511000	0.707108000
1	2.309713000	0.602142000	0.558607000
1	-1.185814000	-2.645557000	-0.763190000
1	-2.434231000	-3.881835000	-0.489865000
6	-1.564068000	-3.376025000	-0.034675000
6	-0.482859000	-4.395164000	0.297886000
1	-0.893185000	-5.191131000	0.931568000
1	-0.119135000	-4.877077000	-0.624724000
8	-0.303027000	-2.682552000	4.049080000
8	0.302020000	-1.573025000	4.993248000
1	-0.119824000	-1.792639000	5.851010000
1	0.804228000	-2.498313000	4.642906000

^{2}F1

E(UM06L) = -1040.39364772

29	-1.007214000	-1.234504000	2.377223000
6	-3.639191000	-2.667944000	2.592492000
1	-3.052805000	-3.540798000	2.905426000
1	-3.821526000	-2.050211000	3.480586000
1	-4.604249000	-3.015481000	2.180286000
6	-3.636617000	-0.657704000	1.209454000
6	-2.680266000	0.492710000	0.934136000
1	-2.062367000	0.260241000	0.055459000
6	-2.473364000	1.224087000	3.268435000
1	-3.227254000	0.501978000	3.604822000
1	-1.752104000	1.358554000	4.082547000
1	-2.966774000	2.189054000	3.052359000
6	1.646569000	0.013302000	3.064349000
1	1.832529000	-0.872151000	3.685817000
1	2.613147000	0.465969000	2.777145000
1	1.076645000	0.731833000	3.663513000
6	1.621089000	-1.398716000	1.081774000
1	2.242592000	-0.911743000	0.311387000
1	2.308906000	-1.922380000	1.757034000
6	0.668870000	-2.389850000	0.433295000
1	1.241330000	-3.186046000	-0.076700000
1	0.059331000	-1.880677000	-0.325900000
7	-0.254900000	-2.974095000	1.435113000
7	-2.892374000	-1.878966000	1.602411000
7	-1.768044000	0.711183000	2.083529000
7	0.878888000	-0.386634000	1.874092000
1	-4.320519000	-0.396267000	2.026693000
1	-4.262640000	-0.847948000	0.321146000
1	-3.247763000	1.414317000	0.710131000
6	0.431590000	-3.852045000	2.395033000
1	-0.299186000	-4.232834000	3.119900000
1	0.914891000	-4.704327000	1.883340000
1	1.192710000	-3.290718000	2.950732000
6	-0.621550000	1.578336000	1.719003000
6	0.480603000	0.775335000	1.044122000
1	0.127396000	0.391853000	0.077136000
1	-0.241784000	2.037761000	2.640563000
1	-0.947119000	2.401772000	1.060202000
1	1.351072000	1.426144000	0.843648000
1	-2.136224000	-1.987013000	-0.349288000
1	-3.363279000	-3.221749000	0.007623000
6	-2.493429000	-2.681348000	0.423781000
6	-1.392737000	-3.667050000	0.784280000
1	-1.774197000	-4.420938000	1.484513000
1	-1.057742000	-4.208031000	-0.117483000
8	-1.184972000	-1.717250000	4.392339000
8	-0.688129000	-0.643233000	5.270482000
1	-1.514467000	-0.234563000	5.572090000

^{2}F2

E(UM06L) = -1040.38338670

29	-1.101903000	-0.866349000	2.610988000
6	-3.712513000	-2.263299000	2.893360000
1	-3.107705000	-3.109124000	3.243428000
1	-3.909043000	-1.610118000	3.753244000
1	-4.671124000	-2.646486000	2.499474000
6	-3.719917000	-0.289504000	1.439446000
6	-2.759952000	0.865836000	1.188012000
1	-2.129808000	0.642408000	0.315384000
6	-2.586612000	1.572113000	3.536556000
1	-3.437806000	0.921682000	3.770538000
1	-1.911381000	1.544964000	4.400014000
1	-2.956533000	2.598863000	3.364887000
6	1.521037000	0.321203000	3.377162000
1	1.765491000	-0.596692000	3.927075000
1	2.454553000	0.862332000	3.138739000
1	0.892352000	0.932699000	4.033779000
6	1.528545000	-1.034414000	1.340205000
1	2.125461000	-0.535521000	0.558882000
1	2.238501000	-1.551701000	1.996901000
6	0.572860000	-2.032780000	0.703602000
1	1.141374000	-2.832712000	0.196063000
1	-0.041830000	-1.527962000	-0.054488000
7	-0.345080000	-2.612313000	1.716478000
7	-2.979433000	-1.504033000	1.868159000
7	-1.865508000	1.067983000	2.355263000
7	0.786172000	-0.036560000	2.152919000
1	-4.436131000	-0.026852000	2.227630000
1	-4.310706000	-0.493040000	0.531096000
1	-3.324138000	1.789801000	0.967506000
6	0.354423000	-3.473943000	2.683819000
1	-0.371972000	-3.864663000	3.408860000
1	0.852460000	-4.321115000	2.178859000
1	1.106924000	-2.897695000	3.237014000
6	-0.706912000	1.936588000	2.033148000
6	0.401174000	1.149467000	1.350642000
1	0.060030000	0.792363000	0.369042000
1	-0.339015000	2.363062000	2.975283000
1	-1.019358000	2.781564000	1.395891000
1	1.276388000	1.801788000	1.179961000
1	-2.238302000	-1.665930000	-0.085330000
1	-3.463200000	-2.889232000	0.315416000
6	-2.588534000	-2.340416000	0.708377000
6	-1.485978000	-3.318519000	1.083268000
1	-1.863456000	-4.058539000	1.800118000
1	-1.154073000	-3.875071000	0.190170000
8	-1.099413000	-1.305989000	4.779203000
8	-0.795507000	-0.186691000	5.711126000
1	-0.340383000	-1.897889000	4.898575000

^{3}G1

E(UM06L) = -1040.22451782

29	-1.026084000	-1.199954000	2.228867000
6	-3.620206000	-2.603529000	2.569136000
1	-3.009877000	-3.458324000	2.885426000
1	-3.800588000	-1.971793000	3.448331000
1	-4.585620000	-2.976360000	2.182915000
6	-3.659922000	-0.588885000	1.178659000
6	-2.709318000	0.567085000	0.896755000
1	-2.132414000	0.362983000	-0.016221000
6	-2.382463000	1.241851000	3.242815000
1	-3.166451000	0.548118000	3.570119000
1	-1.630382000	1.301324000	4.038820000
1	-2.827335000	2.240596000	3.085033000
6	1.555064000	0.007832000	3.100334000
1	1.709417000	-0.872101000	3.737106000
1	2.532998000	0.466518000	2.868757000
1	0.944281000	0.727561000	3.656755000
6	1.637005000	-1.442127000	1.134697000
1	2.300731000	-0.977361000	0.387179000
1	2.283186000	-1.955523000	1.857088000
6	0.708017000	-2.440486000	0.457693000
1	1.293631000	-3.270758000	0.025116000
1	0.164835000	-1.951310000	-0.362803000
7	-0.303458000	-2.960055000	1.412946000
7	-2.908536000	-1.815330000	1.549414000
7	-1.744156000	0.747346000	2.011400000
7	0.864104000	-0.403695000	1.867062000
1	-4.329616000	-0.337033000	2.010395000
1	-4.299038000	-0.774845000	0.299910000
1	-3.282851000	1.496213000	0.730609000
6	0.279773000	-3.823456000	2.454162000
1	-0.511748000	-4.125445000	3.151867000
1	0.742181000	-4.724440000	2.013321000
1	1.040744000	-3.274505000	3.022377000
6	-0.587214000	1.592451000	1.616655000
6	0.518688000	0.749371000	0.995270000
1	0.186058000	0.345127000	0.029110000
1	-0.216745000	2.101203000	2.515226000
1	-0.902630000	2.379237000	0.911924000
1	1.411410000	1.372062000	0.808890000
1	-2.159619000	-1.923335000	-0.404611000
1	-3.405521000	-3.144237000	-0.049730000
6	-2.525196000	-2.620096000	0.362877000
6	-1.436597000	-3.625482000	0.716074000
1	-1.836079000	-4.399109000	1.383282000
1	-1.085942000	-4.138767000	-0.194240000
8	-1.236940000	-1.688935000	4.501650000
8	-0.689331000	-0.787005000	5.276383000
1	-0.856873000	-1.071380000	6.205268000

^{3}G2

E(UM06L) = -1040.22131412

29	-1.107581000	-0.847313000	2.464446000
6	-3.650747000	-2.287374000	2.891328000
1	-3.020644000	-3.130250000	3.200937000
1	-3.820271000	-1.649309000	3.768140000
1	-4.618958000	-2.677155000	2.530559000
6	-3.752452000	-0.284865000	1.479493000
6	-2.827619000	0.896013000	1.205360000
1	-2.253655000	0.714979000	0.285314000
6	-2.491611000	1.535530000	3.562657000
1	-3.274786000	0.831464000	3.868875000
1	-1.738838000	1.567295000	4.359187000
1	-2.938156000	2.537670000	3.435961000
6	1.437146000	0.363761000	3.401099000
1	1.612314000	-0.531052000	4.011078000
1	2.402062000	0.859913000	3.194900000
1	0.799506000	1.045866000	3.975687000
6	1.557062000	-1.044729000	1.400403000
1	2.200086000	-0.559118000	0.648588000
1	2.224001000	-1.548224000	2.110549000
6	0.641355000	-2.058257000	0.724805000
1	1.238149000	-2.876258000	0.284805000
1	0.082228000	-1.575174000	-0.088903000
7	-0.353540000	-2.600690000	1.686736000
7	-2.973994000	-1.498255000	1.847769000
7	-1.855611000	1.079901000	2.314711000
7	0.768720000	-0.028745000	2.149701000
1	-4.430966000	-0.051895000	2.309333000
1	-4.382978000	-0.481740000	0.597470000
1	-3.421515000	1.814967000	1.057077000
6	0.257406000	-3.458371000	2.715869000
1	-0.518805000	-3.775197000	3.425455000
1	0.722033000	-4.353351000	2.265897000
1	1.025806000	-2.903587000	3.269392000
6	-0.713924000	1.951253000	1.927893000
6	0.409863000	1.136633000	1.298040000
1	0.088714000	0.745050000	0.322567000
1	-0.354400000	2.458629000	2.831696000
1	-1.045363000	2.737588000	1.230381000
1	1.293529000	1.776522000	1.129018000
1	-2.258102000	-1.609550000	-0.118346000
1	-3.474026000	-2.851407000	0.269159000
6	-2.596641000	-2.307311000	0.660665000
6	-1.481880000	-3.288955000	1.001414000
1	-1.857457000	-4.070246000	1.673606000
1	-1.130746000	-3.794600000	0.087298000
8	-1.043592000	-1.288016000	4.894128000
8	-0.692540000	-0.336390000	5.730637000
1	-0.374064000	-2.006088000	4.972910000

^{3}H

E(UM06L) = -1039.77156496

29	-1.016165000	-1.219406000	2.302051000
6	-3.592493000	-2.695299000	2.588428000
1	-2.978001000	-3.555814000	2.879421000
1	-3.773160000	-2.091804000	3.486435000
1	-4.555416000	-3.060532000	2.188482000
6	-3.665997000	-0.659735000	1.244378000
6	-2.749321000	0.520569000	0.954772000
1	-2.183616000	0.338915000	0.030372000
6	-2.393697000	1.211847000	3.293255000
1	-3.212983000	0.549254000	3.596590000
1	-1.644559000	1.203370000	4.095155000
1	-2.787301000	2.235238000	3.159900000
6	1.593433000	0.077899000	3.070710000
1	1.764595000	-0.775244000	3.738227000
1	2.562913000	0.526932000	2.789194000
1	1.000887000	0.818402000	3.619259000
6	1.644216000	-1.410196000	1.144987000
1	2.309773000	-0.939312000	0.402174000
1	2.289024000	-1.919698000	1.871713000
6	0.729543000	-2.416086000	0.463267000
1	1.330135000	-3.225954000	0.011579000
1	0.169392000	-1.927578000	-0.346025000
7	-0.257118000	-2.972687000	1.420495000
7	-2.884532000	-1.873434000	1.594368000
7	-1.771505000	0.718135000	2.053272000
7	0.863895000	-0.378825000	1.875919000
1	-4.325001000	-0.427868000	2.090369000
1	-4.317776000	-0.853506000	0.376145000
1	-3.349070000	1.435813000	0.803648000
6	0.357341000	-3.849340000	2.429936000
1	-0.412383000	-4.166419000	3.144762000
1	0.814529000	-4.739666000	1.961933000
1	1.128108000	-3.304521000	2.988950000
6	-0.634583000	1.577912000	1.635840000
6	0.470387000	0.749708000	0.994487000
1	0.119940000	0.324394000	0.043838000
1	-0.254244000	2.091423000	2.527608000
1	-0.975501000	2.360413000	0.937803000
1	1.343571000	1.388943000	0.772767000
1	-2.133067000	-1.930222000	-0.362118000
1	-3.359636000	-3.175612000	-0.032687000
6	-2.487630000	-2.646707000	0.392003000
6	-1.385160000	-3.642554000	0.725494000
1	-1.771817000	-4.428430000	1.386267000
1	-1.036188000	-4.142506000	-0.193484000
8	-1.242485000	-1.668892000	4.417070000
8	-0.523438000	-0.895543000	5.228973000

^{4}I

E(UM06L) = -1039.63060364

29	-1.002636000	-1.191355000	2.095669000
6	-3.533349000	-2.648135000	2.632333000
1	-2.900020000	-3.493440000	2.927664000
1	-3.686205000	-2.011544000	3.513267000
1	-4.508557000	-3.034629000	2.287189000
6	-3.663884000	-0.645442000	1.222087000
6	-2.748973000	0.540920000	0.930245000
1	-2.195694000	0.365916000	-0.004006000
6	-2.351445000	1.160085000	3.284894000
1	-3.092067000	0.425679000	3.624191000
1	-1.567847000	1.230905000	4.049227000
1	-2.842760000	2.142864000	3.173948000
6	1.488485000	-0.003133000	3.094491000
1	1.665610000	-0.908331000	3.688922000
1	2.447635000	0.518004000	2.927321000
1	0.819910000	0.651441000	3.666296000
6	1.660379000	-1.399646000	1.079878000
1	2.297159000	-0.918044000	0.320761000
1	2.333007000	-1.887301000	1.795862000
6	0.751749000	-2.431794000	0.417898000
1	1.353952000	-3.258516000	0.002468000
1	0.199092000	-1.966925000	-0.411166000
7	-0.254146000	-2.952309000	1.380601000
7	-2.876050000	-1.858741000	1.578009000
7	-1.747729000	0.725181000	2.013912000
7	0.861256000	-0.382170000	1.817221000
1	-4.327892000	-0.414228000	2.063995000
1	-4.308724000	-0.844333000	0.351078000
1	-3.350085000	1.457664000	0.799294000
6	0.337934000	-3.778327000	2.447107000
1	-0.451320000	-4.070615000	3.151502000
1	0.811563000	-4.684932000	2.031318000
1	1.091132000	-3.204007000	3.000386000
6	-0.617711000	1.602378000	1.599477000
6	0.508240000	0.786912000	0.968862000
1	0.187325000	0.396822000	-0.007782000
1	-0.252967000	2.129424000	2.489699000
1	-0.964040000	2.372266000	0.891446000
1	1.393133000	1.426181000	0.804462000
1	-2.186850000	-1.968599000	-0.398971000
1	-3.394380000	-3.215102000	0.004289000
6	-2.513545000	-2.668356000	0.383630000
6	-1.386589000	-3.645741000	0.704746000
1	-1.747999000	-4.435066000	1.375155000
1	-1.044314000	-4.141309000	-0.217802000
8	-1.160843000	-1.838607000	4.828298000
8	-0.711099000	-0.896188000	5.418609000
